# Gene connectivity and enzyme evolution in the human metabolic network

**DOI:** 10.1186/s13062-019-0248-7

**Published:** 2019-09-03

**Authors:** Begoña Dobon, Ludovica Montanucci, Juli Peretó, Jaume Bertranpetit, Hafid Laayouni

**Affiliations:** 1 Institut de Biologia Evolutiva (UPF-CSIC), Universitat Pompeu Fabra, Dr. Aiguader 88, 08003 Barcelona, Catalonia Spain; 2 Dipartimento di Biomedicina Comparata e Alimentazione, Università degli Studi di Padova, Padua, Italy; 3 Institute for Integrative Systems Biology I2SysBio (University of Valencia-CSIC) and Department of Biochemistry and Molecular Biology, University of Valencia, Valencia, Spain; 4 Bioinformatics Studies, ESCI-UPF, Pg.Pujades 1, 08003 Barcelona, Catalonia Spain

**Keywords:** Network topology, Degree, Connectivity, Metabolism, Enzymes, Positive selection, Purifying selection

## Abstract

**Background:**

Determining the factors involved in the likelihood of a gene being under adaptive selection is still a challenging goal in Evolutionary Biology. Here, we perform an evolutionary analysis of the human metabolic genes to explore the associations between network structure and the presence and strength of natural selection in the genes whose products are involved in metabolism. Purifying and positive selection are estimated at interspecific (among mammals) and intraspecific (among human populations) levels, and the connections between enzymatic reactions are differentiated between incoming (in-degree) and outgoing (out-degree) links.

**Results:**

We confirm that purifying selection has been stronger in highly connected genes. Long-term positive selection has targeted poorly connected enzymes, whereas short-term positive selection has targeted different enzymes depending on whether the selective sweep has reached fixation in the population: genes under a complete selective sweep are poorly connected, whereas those under an incomplete selective sweep have high out-degree connectivity. The last steps of pathways are more conserved due to stronger purifying selection, with long-term positive selection targeting preferentially enzymes that catalyze the first steps. However, short-term positive selection has targeted enzymes that catalyze the last steps in the metabolic network. Strong signals of positive selection have been found for metabolic processes involved in lipid transport and membrane fluidity and permeability.

**Conclusions:**

Our analysis highlights the importance of analyzing the same biological system at different evolutionary timescales to understand the evolution of metabolic genes and of distinguishing between incoming and outgoing links in a metabolic network. Short-term positive selection has targeted enzymes with a different connectivity profile depending on the completeness of the selective sweep, while long-term positive selection has targeted genes with fewer connections that code for enzymes that catalyze the first steps in the network.

**Reviewers:**

This article was reviewed by Diamantis Sellis and Brandon Invergo.

**Electronic supplementary material:**

The online version of this article (10.1186/s13062-019-0248-7) contains supplementary material, which is available to authorized users.

## Background

Proteins are not independent entities, but part of complex biomolecular interacting networks. Previous studies have analyzed the relation between network structure and gene evolution in different phylogenetic groups by analyzing their divergence based on synonymous and nonsynonymous changes in exons. Most of the studies focused on the effect of purifying selection on gene evolution, showing a trend shared by metabolic networks, protein-protein interaction networks (PIN), and individual pathways from different organisms: purifying selection is stronger in highly connected and more central genes [1–5]. Conversely, the constraints imposed by the position of the enzyme along the pathway seem organism-specific or system-specific: purifying selection is stronger in upstream genes of plant biosynthetic pathways [6, 7] and human metabolic pathways [5], but, in animals, downstream genes of the Insulin/TOR signal transduction pathway are more constrained than upstream genes [8, 9]. Divergence data has also been used to measure which parts of the network are more prone to be under long-term positive (adaptive) selection. As with purifying selection, some features seem to be shared across organisms, whereas others appear lineage specific. Positive selection has acted preferentially in genes coding for enzymes at branch points in *Drosophila* and humans [10, 11]. Positive selection has also acted in peripheral genes in the human [4, 12] and yeast PIN [13]. Remarkably, the same study found the opposite trend in the *Drosophila* PIN: positive selection was detected mostly in central genes [13].

Few studies have used both divergence (interspecific) and polymorphism (intraspecific) information to infer the strength of positive and negative selection in large-scale networks. While long-term positive selection has acted in the periphery of the human PIN, short-term positive selection, as detected by polymorphism data, has acted in more central genes [4, 14]. A similar result was observed in the Insulin/TOR signal transduction pathway [15]. These studies, which analyzed either small pathways or PINs, are of complex interpretation. They showed the need for studying the relationship between positive and purifying selection and network topology at different evolutionary timescales to unravel where and how natural selection acts in a biomolecular network. The present study aims to test the previous results on the best known and curated cellular network, the metabolic network, and explore its particularities.

Metabolism is one of the best described cellular systems, comprising a complex universe of reactions on which we can study the action of natural selection. The application of network theory can discover the evolutionary constraints (purifying selection) or the evolutionary innovations (positive selection) imposed on enzyme-coding genes by the intrinsic structure of the network. Here, we have performed an evolutionary analysis of the human metabolic network from a top-down approach: from the whole metabolic network to individual metabolic pathways. We have analyzed the presence and strength of natural selection at two levels: interspecific, among mammals (during the divergence of primates and rodents), and intraspecific, at the level of human populations. Our goal is to establish where both, purifying and adaptive selection, have been acting in the metabolic network and to determine the role of topology in shaping the evolution of enzyme-coding genes. We aim to answer the following question: given a complex metabolic network, which parts will be more constrained during its evolution, and where will the innovations happen based on the connections between the gene products?

## Results

We represented the human metabolic network as a directed reaction graph, where nodes are enzymatic reactions, and consequently are associated to the genes that code for the enzymes performing that reaction (see Additional file 1: Figure S1, and Additional file 2: Table S1). Nodes are linked by shared metabolites: if the product of an enzymatic reaction is the substrate of another, then a directed link is generated between the nodes representing the reactions. The number of connections or links of an enzymatic reaction are separated in: incoming links (in-degree), representing the number of reactions that produce the metabolites that our reaction accepts as substrates, and outgoing links (out-degree), representing the number of reactions that use as substrates the products of our reaction. This reaction-graph representation was applied to two datasets: to the latest genome-scale network reconstruction of the human metabolism, Recon3D [16], and to individual metabolic pathways from HumanCyc Pathway/Genome database [5, 17]. The choice of these two sources was motivated by the problem of how to define a metabolic pathway and its boundaries. A large-scale network will allow us to infer global patterns and account for crosstalk effects between biological processes, with the drawback that the interactions may be less reliable given that considerable information was computationally driven and compliant for metabolic modelling. Therefore, metabolic reactions without genetic evidence but with physiological evidence or required for modeling are included with different confidence scores [18]. On the other side, comparing hundreds of small-scale networks might allow us to uncover local shared patterns with an easier biological interpretation. A dimension not covered in the present study is the differences due to tissue-specific expression or to a given developmental stage. As we are using a general model of the metabolism and not a cell-specific model, the dynamics of the system are not considered here, even when it is known that genes encoding enzymes with high metabolic fluxes have been more constrained in their evolution [19]. Our approach reveals the overall, stratified effects of selection forces potentially acting at different times or tissues. For this reason, it is not able to reveal evolutionary patterns that are specific to a tissue or to a developmental stage and may make more difficult to interpret the results and identify the specific biological function under selection.

### Purifying selection in mammals is stronger in highly connected nodes

**Fig. 1:**
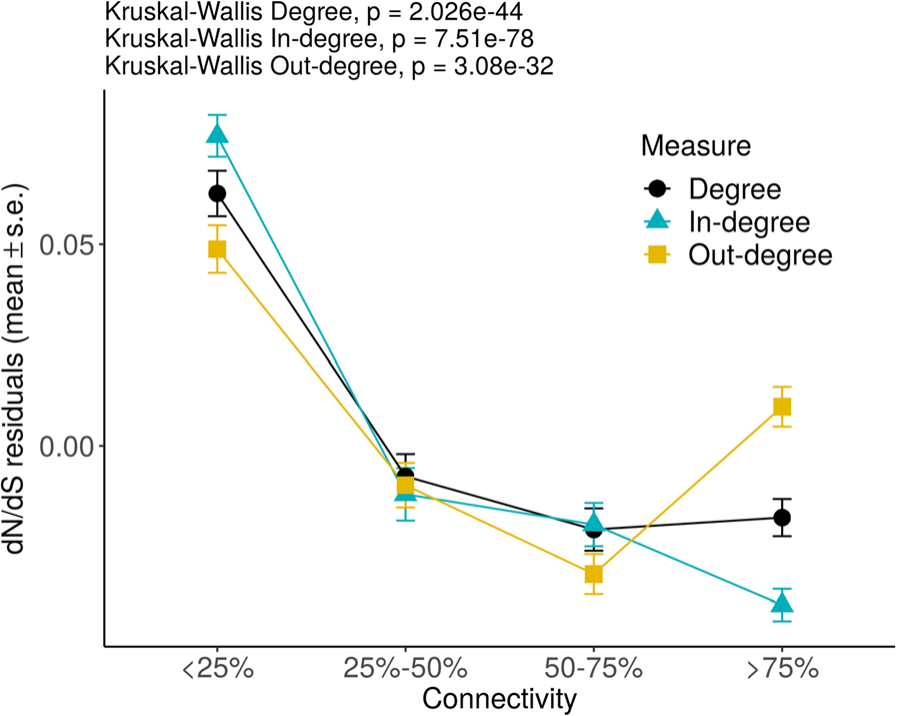
Strength of purifying selection estimated among mammals versus gene connectivity in the human metabolic network. Nodes were divided using the 25th, 50th, and 75th percentiles and the mean ± standard error of the residuals of a linear regression of dN/dS controlling for genomic variables (CDS length, codon bias, and GC content) is plotted for each group. Global differences between groups were assessed by Kruskal-Wallis Rank Sum test. Highly connected genes are under stronger purifying selection

The strength of purifying selection in the global metabolic network was measured as the ratio between the rate of nonsynonymous substitutions (dN) and the rate of synonymous substitutions (dS), where lower values of dN/dS indicate stronger purifying selection. Most enzyme-coding genes have a dN/dS value lower than 0.5, indicating the widespread action of purifying selection in metabolic genes (see Additional file 1: Figure S2). The possible effect of confounding genomic variables has been considered (see Additional file 1: Figure S3) by applying a linear regression on the evolutionary estimates controlling for protein-coding sequence (CDS) length, GC content, and codon bias, and using the residuals values instead of the original scores. After removing the effect of the confounding variables, we find that purifying selection is stronger in nodes with more connections (Fig. 1 and Additional file 1: Figure S4a). Interestingly, nodes with extremely high out-degree are less constrained due to decreasing values of dS (see Additional file 1: Figure S4b-c). As similarly found in individual metabolic pathways [5], genes encoding enzymes that catalyze the first steps in the metabolic network are under weaker purifying selection than those catalyzing reactions in intermediate and final steps (see Additional file 1: Figure S5a).

### Node connectivity influences the action of positive selection

In the global metabolic network, we found 67 genes (3.79% of the metabolic genes) under positive selection among mammals by applying the site model M8 in PAML (M7/M8) to detect selection events in any of the lineages. By applying the branch-site test of positive selection (Test 2 in PAML), we detected nine genes (0.51%) under positive selection in the human lineage (see Additional file 2: Table S2). Genes under positive selection among mammals show different connectivity than the rest of the metabolic genes: they encode enzymes with low connectivity, with both lower in-degree and out-degree than the metabolic genes without evidence of positive selection (Table 1). Similarly, genes selected only in the human lineage show lower out-degree than the neutral genes. Also based on their connectivity, we classified the position of the nodes within the network: genes under positive selection among mammals are found preferentially at top positions (in-degree = 0) (Pearson’s Chi-squared test, Χ^2^ = 1200, *p*-value = 0.0005; Additional file 1: Figure S5d). Thus, long-term positive selection has acted preferentially on poorly connected or peripheral genes associated with the first steps of metabolic processes.

**Table 1:** Connectivity of metabolic genes under positive selection compared to the rest of metabolic genes of the global metabolic network

Test	Connectivity	Ng	Nr	Mean	Sampling mean	*p*-value
M7/M8	Degree	67	707	0.0016	0.0063	**< 0.0001**
Test 2	Degree	9	99	0.0061	0.0063	0.8382
CEU Complete	Degree	13	27	0.0010	0.0055	**0.0044**
CHB Complete	Degree	22	238	0.0017	0.0055	**< 0.0001**
CEU Incomplete	Degree	19	100	0.0097	0.0055	**< 0.0001**
CHB Incomplete	Degree	15	143	0.0076	0.0055	**0.0015**
M7/M8	In-degree	67	707	0.0008	0.0035	**< 0.0001**
Test 2	In-degree	9	99	0.0044	0.0035	0.1312
CEU Complete	In-degree	13	27	0.0005	0.0029	**0.0210**
CHB Complete	In-degree	22	238	0.0007	0.0029	**< 0.0001**
CEU Incomplete	In-degree	19	100	0.0008	0.0030	**0.0004**
CHB Incomplete	In-degree	15	143	0.0011	0.0029	**0.0004**
M7/M8	Out-degree	67	707	0.0007	0.0028	**< 0.0001**
Test 2	Out-degree	9	99	0.0018	0.0028	**0.022**
CEU Complete	Out-degree	13	27	0.0004	0.0026	**0.0174**
CHB Complete	Out-degree	22	238	0.0010	0.0026	**< 0.0001**
CEU Incomplete	Out-degree	19	100	0.0090	0.0026	**< 0.0001**
CHB Incomplete	Out-degree	15	143	0.0065	0.0026	**< 0.0001**

Ng: number of genes in the global metabolic network under positive selection in the mammalian lineage (M7/M8), the human lineage (Test 2) or in recent human evolution (Incomplete and Complete selective sweeps in Europeans (CEU) and Asian (CHB) populations); Nr: number of enzymatic reactions coded by the genes under positive selection; Mean: mean connectivity value of the genes under positive selection; Sampling mean: mean connectivity value of the sampling distribution based on all metabolic genes; *p*-value: two-sided exact p-value calculated using 10,000 Monte Carlo simulations. *P*-values < 0.05 are highlighted in bold.

To detect recent positive selection in human populations, we used the Hierarchical Boosting (HB) [20] to detect genes under complete (Complete HB) and incomplete selective sweeps (Incomplete HB). In human populations, out of the 1769 genes encoding enzymes in the global metabolic network, we found under positive selection in Europeans (CEU) 13 genes with a complete selective sweep (0.73% of metabolic genes) and 19 genes with an incomplete sweep (1.07%), and in Asians (CHB) 22 genes with a complete (1.24%) and 15 genes with an incomplete selective sweep (0.85%) (see Additional file 2: Table S2). No signal of positive selection was found in metabolic genes in the Sub-Saharan African population (YRI), but that is expected given the low number of signals detected by the Hierarchical Boosting in YRI [20]. Metabolic genes under positive selection in humans (both in CEU and in CHB) show different connectivity than the rest of enzyme-coding genes (Table 1). Genes under a complete selective sweep encode for poorly connected enzymes, with both lower in-degree and out-degree than the rest of metabolic genes. But genes under an incomplete selective sweep show a different connectivity pattern: even though they still code for enzymes with lower in-degree, they have higher out-degree than the average metabolic gene. Thus, genes under a complete selective sweep behave similar to those detected under long-term positive selection, whereas those under an incomplete sweep are highly connected by outgoing links. The action of recent positive selection among human populations varies depending on the final frequency of the selected variant.

When looking at the strength of recent positive selection in relation to connectivity, the pattern is complex (see Additional file 1: Figure S4d-g). Genes with low connectivity tend to have smaller values of HB than genes with higher connectivity, except in the complete HB in CEU, where genes with high out-degree have very low HB values. Regarding the position of the node within the network, there is a clear linear trend in CEU. Genes involved in the first steps in the metabolic network have lower values of the HB (Complete and Incomplete) than genes participating in intermediate and bottom steps, with genes associated with the last steps having the highest values. We do not observe this trend in CHB. Genes participating in intermediate and last steps do have higher values of HB Complete than genes performing the first steps, but there is no difference between the intermediate and bottom categories. There is no significant difference between values of HB Incomplete in CHB depending on the position of the gene within the pathway (see Additional file 1: Figure S5b). Accordingly, we only find differences in the number of genes under recent positive selection according to node position in CEU: both, genes under complete or incomplete selective sweeps code for enzymes that act in the last steps of the metabolic network (Pearson’s Chi-squared test, *p*-value < 0.05, see Additional file 1: Figure S5d).

In the smaller dataset of individual metabolic pathways, we detected in CEU three genes with a complete selective sweep (0.32% of the metabolic genes in individual pathways) and 10 genes with an incomplete sweep (1.06%). In CHB, we found 11 genes with a complete (1.16%), and nine genes with an incomplete selective sweep (0.95%) (see Additional file 2: Table S3). Only genes under an incomplete selective sweep in CHB show lower value of in-degree than the rest of metabolic genes (see Additional file 2: Table S4). We see a similar trend in CEU both in the individual metabolic pathways and in the global network: genes at top positions have smaller values of the complete HB than genes at intermediate or bottom positions (see Additional file 1: Figure S5c). However, we do not find differences in the number of genes under positive selection according to node position.

### Not all metabolic functions are under the same selective pressures

**Fig. 2:**
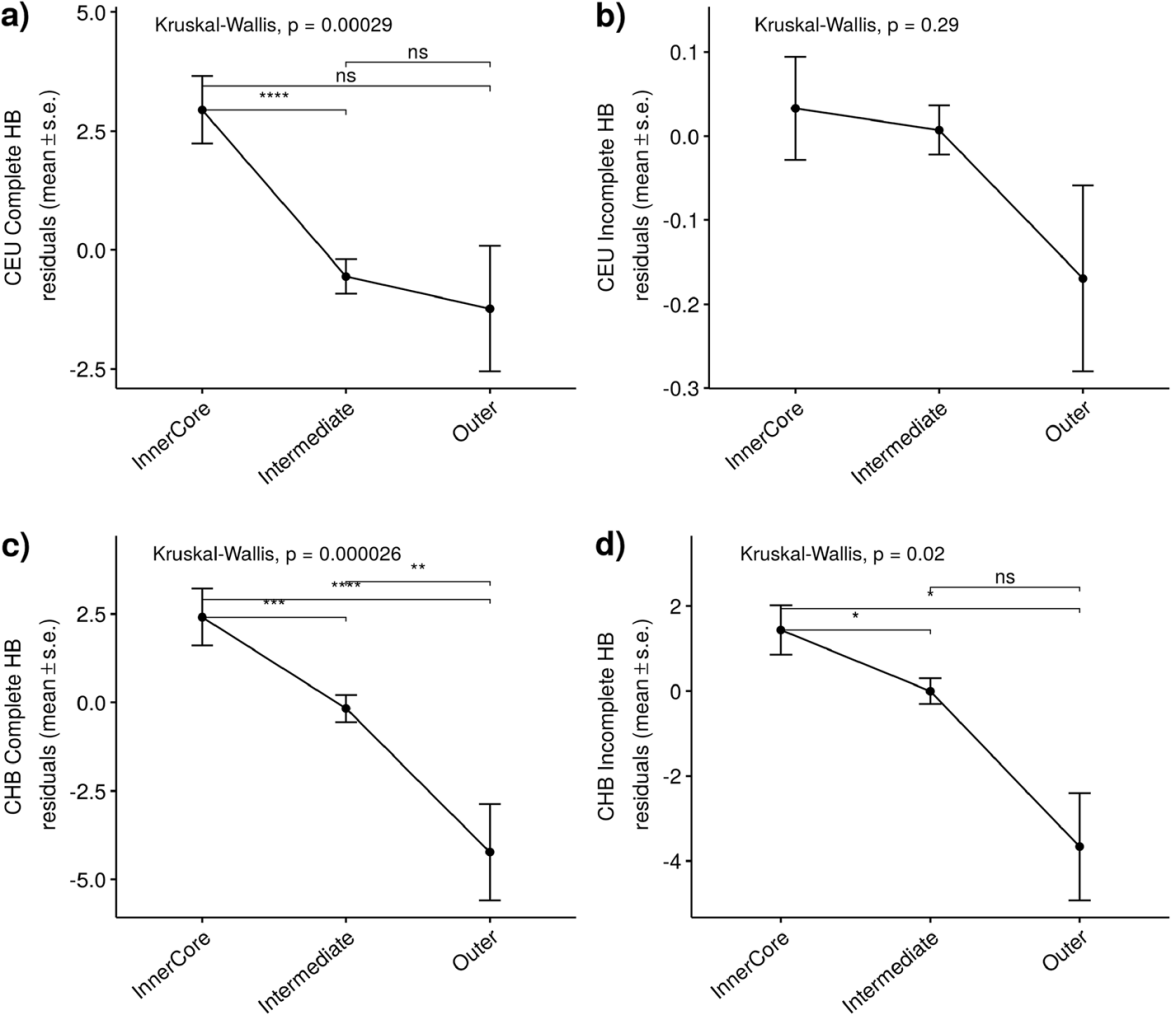
Relationship between recent selection in humans and metabolic functions. Individual metabolic pathways were classified based on a global view of the metabolism as a three-layered system as described in [5]. Mean ± standard error of the residuals of a linear regression of the Hierarchical Boosting (HB) scores controlling for genomic variables (CDS length, codon bias, and GC content) is plotted for each category. **a**) Complete HB scores in CEU, **b**) Incomplete HB scores in CEU, **c**) Complete HB scores in CHB, and **d**) Incomplete HB scores in CHB. Inner Core: Glycolysis / Tricarboxylic Acid Cycle / Pentose Phosphate and Polysaccharides; Intermediate: Membrane Lipids, Nucleotide, Fatty Acid / Triacylglyceride, Cofactor, Fatty Acid / Hormone, and Amino acid; Outer: Steroid, Secondary Metabolism and Detoxification. Pair-wise *p*-values are adjusted by FDR (ns: *p* > 0.05; *: *p* < = 0.05; **: *p* < = 0.01; ***: *p* < = 0.001; ****: *p* < = 0.0001)

Individual metabolic pathways can be grouped according to their main metabolic function based on a global view of the metabolism as a three-layered system [5]: i) Inner Core (Glycolysis / Tricarboxylic Acid Cycle / Pentose Phosphate and Polysaccharides), ii) Intermediate (Membrane Lipids, Nucleotide, Fatty Acid / Triacylglyceride, Cofactor, Fatty Acid / Hormone, and Amino acid) and iii) Outer (Steroid, Secondary Metabolism and Detoxification). We compared differences in evolutionary measures between groups (Fig. 2). Pathways belonging to the inner core have higher values of HB scores than the other layers, with a stronger trend in Complete HB. However, we only find differences in the number of genes under positive selection among categories in CHB, where there are more genes than expected under an incomplete selective sweep in the intermediate and outer layers (Pearson’s Chi-squared test, Χ^2^ = 6.6, *p*-value = 0.04).

In a similar way as for individual pathways, we calculated whether there is any functional pathway within the global network enriched in genes under positive selection. Metabolic functions related to lipid metabolism (fatty acid oxidation, glycerophospholipid metabolism, cholesterol and bile acid metabolism) and to membrane transport are enriched with positively selected genes (Pearson’s Chi-squared test, p-value < 0.05 in all tests, see Additional file 1: Figure S6). All these processes are functionally interconnected since they are involved in lipid transport and utilization as well as membrane fluidity and permeability.

As expected, there is no one-to-one mapping between genes and enzymatic reactions in the metabolic network: 61.60% of the genes encode for enzymes that participate in more than one reaction, and on average, a gene participates in 7.44 reactions (see Additional file 1: Figure S7). The number of functions of a gene or the number of enzymatic reactions carried out by the enzyme(s) coded by the gene is a measure of molecular gene pleiotropy [21]. When comparing the genes under positive selection to the rest of metabolic genes, we do not find differences in the number of enzymatic reactions performed by enzymes coded by positively selected genes, either at inter or intraspecific level (Permutation test, *p*-value > 0.05 in all comparisons).

## Discussion and conclusions

With this study, we add evidence that the structure of the metabolic network matters for the action of natural selection, both in its constraints through purifying selection and in the production of innovations through positive selection.

Purifying selection is stronger in highly connected genes, as previously described in the metabolic networks [2, 3, 22] and PINs [1, 4] of other organisms. This reinforces the converging evidence of stronger conservation for genes whose products are important in terms of connectivity. We have also confirmed using the most recent metabolic reconstruction that in the metabolism, the generation of the end-product is more preserved than the initial steps of the pathways [5]: genes catalyzing the last steps of the pathways are more conserved than genes catalyzing the first steps (see Additional file 1: Figure S5a).

The distribution of positive selection in the metabolic network is complex: at the interspecific level and in the complete selective events within humans, new adaptations appear in genes whose products are poorly connected in the network. Whereas in the case of ongoing positive selection (incomplete sweeps), it is detected in genes highly connected by outgoing links: genes that code for enzymes whose products are used by many other reactions.

Long-term positive selection has targeted genes that code for enzymes that catalyze the first steps of the metabolic network, supporting the idea that the generation of the end-product is more preserved in the metabolism. However, short-term selection, as indicated by the complete and incomplete Hierarchical Boosting, has targeted preferentially enzymes that catalyze the last steps of the metabolic network (see Additional file 1: Figure S5d).

Even though recent positive selection is stronger in the inner core of the metabolism (Fig. 2), pointing to higher adaptation in genes that participate in central metabolic pathways, there is not a strong difference in the number of genes under positive selection among the different layers. Only the intermediate and outer layers show more genes under an incomplete selective sweep in CHB than expected. The intermediate and outer layer comprise, among others, pathways related to membrane lipids and fatty acid metabolism. When looking at the global network, similar functionally related pathways (lipid metabolism and membrane transport) are enriched in positively selected genes (see Additional file 1: Figure S6), suggesting that these metabolic processes have been targets of positive selection at both inter and intraspecific level. Recent selection in metabolic pathways related to carbohydrate, lipid and transport metabolism has been associated with dietary changes in humans due to the Neolithic transition in the last 10 Kya (thousand years ago) [23]. The increased availability of grain-based products and therefore, the increased consumption of carbohydrate-rich foods is a very recent modification of the human diet [24]. The Hierarchical Boosting was calibrated using different selection scenarios with selective events occurring in the range of 45 to 10 Kya [20]. Thus, this very recent change on the diet happened on the limit of the range of the simulations used in the method. This could explain that we do not detect more genes under positive selection in pathways of the inner core of the metabolism that include carbohydrate metabolism (glycolysis and pentose phosphate and polysaccharides) despite the high HB values.

It is difficult to know how other findings exactly relate to ours, as previous studies in whole metabolic networks did not differentiate between incoming (in-degree) and outgoing (out-degree) links [2, 22, 25]. A highly connected gene in an undirected network could translate in a directed network into several ways: a highly connected gene by incoming links (high in-degree), a highly connected gene by outgoing links (high out-degree), or a gene with both high in-degree and high out-degree. Therefore, the overall picture is not simple, but a general pattern emerges: the network structure of the metabolism influences the opportunities of natural selection to act. The constraint imposed by purifying selection is stronger in highly connected genes, and in the last steps of pathways even if the number of reactions in which a gene participates does not restrict it [25, 26].

Adaptive selection follows a pattern close to that observed in the human PIN [4]: long-term positive selection has acted in peripheral genes, but very recent ongoing selection is seen in central genes, specifically highly connected genes by outgoing links. These results can be interpreted by considering the hierarchal structure of metabolic pathways, where upstream or highly connected genes are expected to have far-reaching effects on the overall metabolism than downstream or poorly connected genes [27]. Under Fisher’s Geometric Model of Adaptation (FGM) [28] as the phenotypic complexity of an organism increases, it will be less likely that a mutation is beneficial, as not all traits (or dimensions in the phenotypic space) can be optimized at the same time. Therefore, only mutations with small effects will be more likely to be beneficial. However, this changes if the organism is far from the optimum fitness. Mutations with large effects are more likely to be beneficial if an organism is far from the optimum [27–29]. The pattern found by the Hierarchical Boosting agrees with a species that has been far from the optimum at several times during their recent evolution (out-of-Africa and the Mesolithic-Neolithic transition [30, 31]), generating this result of strong complete selective events in genes with smaller effects (low connectivity) and incomplete selective events in genes with larger effects in the phenotype (higher outgoing links). Thus, the relationship between the action of adaptive selection and gene connectivity depends on the type of positive selection and the evolutionary timescale considered [4]. This seems to be the most remarkable trait of the evolvability of biomolecular networks.

## Methods

### Reaction graphs of the human metabolic network and metabolic pathways

We have obtained information of enzyme-coding genes of the human metabolic network from two sources. The first dataset corresponds to the most comprehensive human metabolic network reconstruction (Recon3D) [16]. It was downloaded from https://vmh.uni.lu in MATLAB format, read using COBRApy Python package [32] and transformed into a directed reaction graph [33]. In a reaction graph, nodes represent enzymatic reactions and by extension, the genes that encode the enzymes that catalyze them. We created a directed link between node A (representing an enzymatic reaction) and node B (representing another enzymatic reaction) if the products of node A are substrates of node B. Three types of reactions were excluded when creating the graph: *biomass_reaction*, *biomass_maintenance*, and *biomass_maintenance_noTrTr*. These reactions are different versions of the biomass function reaction generated to create the stoichiometrically consistent flux balance model and do not correspond to real biochemical reactions [16]. The top highly connected metabolites, the so-called currency metabolites (ADP, ATP, CO_2_, O_2_, H_2_O, H_2_O_2_, H, K, NA_1_, NAD, NADH, NADP, NADPH, NH_4_, Pi, and PP_i_), where not used to define the topological structure of the reaction graph to avoid creating a densely connected graph [2, 34]. Indeed, given that each of these metabolites is involved in almost all the reactions, their inclusion would have connected each node with all the remaining nodes, creating links that do not correspond to real biological metabolic routes and hiding the real topology of the network. This procedure generated one giant connected component and 966 small connected components. For our purpose, we restricted our analyses to the giant connected component formed by 9402 reactions, 178,613 links, and encoded by 1769 genes. Gene coordinates, gene Ensembl stable identifiers, and HGNC symbols were downloaded using the R (R Core Team 2017) biomaRt package [35] from Ensembl GRCh37 (release 85) [36] based on EntrezGene identifiers. The second data set corresponds to the enzyme-coding genes present in HumanCyc that are part of base metabolic pathways analyzed in [5]. From it we selected 843 reactions encoded by 915 genes, corresponding to 275 individual metabolic pathways. There are 768 overlapping genes between both datasets.

### Comparative sequences

For each human enzyme-coding gene present in Recon3D, we retrieved its orthologous protein-coding sequences (CDS) in Chimpanzee, Gorilla, Orangutan, Mouse, and Rat from Ensembl (release 85) [36] using the python program EASER (Ensembl Easy Sequence Retriever, version 1.7.0) [37]. Multiple sequence alignments were generated using T-coffee (default options, version 7.95) [38] by creating a protein sequence alignment and back-translating it to DNA sequence. Only human genes with 1:1 orthologs in the five species were used in the analysis. Multiple sequence alignments that covered less than 60% of the human coding sequence were excluded from the estimations of evolutionary rates, resulting in 1158 genes. From the human CDS we calculated the following sequence-related variables: CDS length, GC content, and codon bias with CodonW (version 1.4.2) [39]. The effective number of codons (ENC) was used as a proxy for codon bias.

### Purifying selection during primate and rodent divergence

The strength of purifying selection at protein level was measured by the program codeml (model M0) of PAML 4 [40] as the nonsynonymous/synonymous substitutions rates ratio (dN/dS). Following the procedure in [5], the model was run five times in the multiple sequence alignments, each run with three initial dN/dS values (0.1, 1 and 2), to assess robustness and discard unstable results.

### Positive selection during primates and rodent divergence

To detect positive selection along the mammal lineage we applied two likelihood ratio tests (LRT) between nested models to the multiple sequence alignments: a) M7/M8 (model M8) to detect selection events in any of the lineages, and b) branch-site test of positive selection (Test 2) to detect selection events in the human branch. Both models are implemented in the program codeml of PAML 4 [40] and were run five times, each run with three initial dN/dS values (0.1, 1 and 2) to discard cases of convergence to a local optimum. A gene was considered under positive selection if the *p*-value was lower than 0.05 after correction for multiple testing by False Discovery Rate (FDR) [41].

### Positive selection during recent human evolution

Signatures of positive selection during recent human evolution were obtained from [20] for each enzyme-coding gene in Recon3D and HumanCyc data sets. We extracted the boosting score (Hierarchical Boosting, HB) for the genomic region consisting of 10 kb upstream the transcript starting point to 10 kb past the transcript ending point. HB values differentiate between complete selective sweeps (the selected allele is fixed) and incomplete selective sweeps (selected allele is at high frequency but not fixed). Both, Complete and Incomplete HB, were extracted for the three populations of the 1000 Genomes Project Phase 1: Utah residents with Northern and Western European Ancestry (CEU), Han Chinese in Beijing, China (CHB), and Yoruba from Ibadan, Nigeria (YRI). The maximum value of all windows overlapping a genic region was used as a measure of whether that gene is under positive selection according to the threshold calculated in [20]. HB was calculated only in autosomal chromosomes. The analyses have been done using only CEU and CHB continental populations, as in YRI no metabolic gene was detected to be putatively under positive selection.

In total, 1664 genes from the giant connected component of Recon3D have a value for at least one boosting test in one population: Complete boosting CEU (*n* = 1657), Incomplete boosting CEU (*n* = 1566), Complete boosting CHB (*n* = 1573), Incomplete boosting CHB (*n* = 1573). In the HumanCyc dataset, 915 genes have a value for at least one boosting test in one population: Complete boosting CEU (*n* = 913), Incomplete boosting CEU (*n* = 913), Complete boosting CHB (*n* = 915), Incomplete boosting CHB (*n* = 915).

For comparative purposes, we retrieved the boosting score for all human protein-coding genes in autosomal chromosomes (*n* = 19,214) following the same procedure: Complete boosting CEU (*n* = 17,593), Incomplete boosting CEU (*n* = 17,585), Complete boosting CHB (*n* = 17,677), Incomplete boosting CHB (*n* = 17,677). We found no differences in the proportion of metabolic and non-metabolic genes detected as being under positive selection in any boosting test in any population (Fisher’s Exact test, *p*-value > 0.05 in all cases).

### Network analyses

For each node (enzymatic reaction) of the giant connected component of the global metabolic network we computed its connectivity using the NetworkX Python package [42]: normalized degree, normalized in-degree and normalized out-degree. The position of the nodes within the network was classified based on their connectivity: top (in-degree = 0), bottom (out-degree = 0) or intermediate (in-degree > 0 and out-degree > 0). Values of the same connectivity measures were retrieved for HumanCyc enzymatic reactions [5].

Values of genomic variables (CDS length, GC content, and codon bias), connectivity (degree, in-degree, out-degree), and selection estimates (Complete and Incomplete HB, dN/dS, dS, and dN) are in Additional file 2: Tables S5 for the genes and reactions of the giant connected component (Recon 3D) and in Additional file 2: Tables S6 for the genes and reactions of the individual metabolic pathways (HumanCyc).

### Common topological features of genes under positive selection

To identify common topological features of positively selected genes, we compared their connectivity values with respect to the rest of enzyme-coding genes by a two-sample randomization t-test (function permTS of R package perm) [43] using a Monte Carlo approximation to the exact *p*-value with 10,000 permutations. We also tested for differences in the strength of selective forces (either purifying or positive selection) by dividing the nodes by connectivity using the 25th, 50th, and 75th percentiles (< 25%, 25–50%, 50–75%, > 75%). An enzymatic reaction can be coded by one or more genes, either as an enzymatic complex or by isozymes. Thus, if an enzymatic reaction (node) is encoded by more than one gene, that node will be associated with as many values of the selection metrics as genes is encoded by. Similarly, if the protein encoded by a gene participates in more than one enzymatic reaction, that gene will be associated with as many connectivity measures as reactions it participates in. The effect of sequence-related variables was controlled by applying a linear regression on the evolutionary estimates controlling for CDS length, GC content, and codon bias, and using the residuals instead of the original values. Prior to applying the linear regression evolutionary estimates were transformed to control for lack of heteroscedasticity using the BoxCoxTrans function from the caret R package. If needed a small positive value was added to the original values to avoid negative or zero values. Global differences between groups and pairwise comparisons were assessed by Kruskal-Wallis and Wilcoxon Rank Sum tests respectively and plotted by the R package ggpubr [44].

## Reviewer’s comments

### Reviewer’s report 1

**Diamantis Sellis**

**Reviewer summary:** I find the article well written, very interesting and important not only because of the findings reported but also due to its interesting methodological approach. The authors combine two different types of analyses: metabolic networks and population genetics. These are often studied in isolation and making a lot of simplifying assumptions. I believe such combined approaches are very promising.

### Reviewer comment

Missing dimensions. The authors chose the human metabolic network. This is probably the mostly studied metabolic network but there is a developmental dimension that is not mentioned in the manuscript. In a multicellular species with multiple types of tissues different cells have slight but significant variations. This temporal and spatial dimension is not at all addressed or commented in the paper which could lead to a misunderstanding of where the findings apply. The effect of selection on the metabolic map is summed across very different cell lines and developmental phases making it very hard to interpret in terms of functional effects.

**Author’s response:** *We agree this is an important dimension not considered in our work and it is beyond the original goals of this study. Our purpose here is to illustrate how the integration of different evolutionary and network scales can explain the evolution of a complex system. This initial approach of using a general model of the human metabolism can be further applied to cell-specific reconstructions. Combined with expression data it certainly will help gain insights into the functional changes and the phenotype under selection. This limitation is now explained at the beginning of the Results section.*

### Reviewer comment

Small metabolites: It is not clear to me why the small metabolites where removed from the dataset. Was this for convenience of analysis, e.g. cannot treat ATP and enzymes in the same analysis, or there is a more fundamental principle?

***Author’s response:*** *Here we study the metabolic network as a reaction graph. In a reaction graph nodes represent enzymatic reactions (and the genes that encode the enzymes that catalyze them) and links are established between two reactions (nodes) if the metabolites that are products of the first reaction (node) are taken as substrates by the second reaction (node). Then, metabolites are only used to determine the connections between reactions (nodes). We do not estimate any metric associated with the metabolites, and we are not interested in the specifics of the metabolites further than to determine the connections between the reactions. However, there are the so-called “currency metabolites”, such as ATP, that are involved in a huge number of reactions that are part of unrelated pathways. Had we used ATP to establish links between reactions we would have linked almost all the reactions among themselves, creating “artificial” links and pathways that do not correspond to real biological processes, hiding the real topology of the network. For this reason, it is an established practice to exclude currency metabolites from the reconstruction of the metabolic reaction graph (Vitkup* et al*, 2006; Ma and Zeng, 2003). Given that this was not sufficiently explained in the text, we modified the Methods section to clarify it.*

### Reviewer comment

Finding robustness: Would the results still valid and to what extent if the effect of confounding factors is not completely removed? In page 6, line 12 the authors explore the strength of purifying selection on genes and try to deal with possible confounding factors. It is not clear if the list of factors is considered exhaustive. Also, the linear regression is a simple tool to remove possible effects but also makes a number of assumptions on the type of the effect of the confounding factors. It is not clear to me to what extent the results still hold if the effect of the possible confounding factors is not completely removed.

***Author’s response:*** *Regarding the exhaustiveness of the confounding factors, we selected the principal factors that affect the rate of protein sequence evolution. Gene length and expression level are the major determinants of evolutionary rates (Pál* et al.*, 2001 Genetics; Drummond* et al.*, 2005 Proc. Natl Acad. Sci.). We used codon bias as a proxy for gene expression, as it is known to be positively correlated with protein abundance (Ghaemmaghami* et al.*, 2003 Nature). This list is by no means exhaustive, but it accounts for the main known drivers of protein sequence evolution. Other variables that correlate with evolutionary rates do so in a smaller measure (Zhang and Yang, 2015, Nature Review Genetics). It is difficult to foresee how the results may change if other confounding factors are added. However, considering the current knowledge on the field, it is unlikely that other variables may explain more variation at genomic level than those included here. As in all correlation analysis, caution must be taken to interpret result in a safe way.*

### Reviewer comment

Minor point: I would like to bring to the authors attention two relevant papers that I think they would find interesting:

http://gutengroup.mcb.arizona.edu/wp-content/uploads/Mannakee2016a.pdf and https://onlinelibrary.wiley.com/doi/abs/10.1111/evo.12548. This is not a suggestion to cite the papers.

***Author’s response:*** *We thank the reviewer for pointing out these papers. As mentioned in another section, while it is a factor that affects gene evolution, we think that to analyze metabolic flux dynamics is outside the scope of our paper. The second paper is very interesting and related to another paper where the authors also argue that positive selection targets different parts of the protein-protein interaction network depending on how far from the fitness optimum is the organism (Luisi* et al*, 2015). We have now commented on that hypothesis in the Discussion.*

**Reviewer comments to Authors:** The authors have adequately addressed all the issues raised by the reviewers and I believe the manuscript have considerably improved.

### Reviewer’s report 2

**Brandon Invergo**

**Reviewer summary:** The authors have investigated how the molecular evolution of metabolic enzymes has been influenced by the topology of substrate/product dependencies between them. These dependencies were represented by a network in which nodes are reactions and directed edges indicate the use of a product of one reaction as the substrate of another. The authors thus compared different metrics of molecular evolution against network-topological metrics such as connectivity, centrality and position. As the authors point out, similar approaches have been applied to several different kinds of molecular networks (metabolic, signaling, etc.) at different scales (pathway, proteomic), and at different evolutionary time scales (intraspecific polymorphism and interspecific divergence). The novelty here is an attempt to synthesize the different network scales and evolutionary scales in the context of the metabolic network. The authors show both interesting similarities and differences between these different views. The work has the potential to be the “final say” in topological constraints on molecular evolution of metabolic enzymes, however it is held back by a lack of synthesis of the various network results with the underlying biology. There are also some potentially serious statistical issues that must be addressed.

### Reviewer comment

The relationship between the present manuscript and the authors’ previous publication (Montanucci et al. 2018. PLoS One) must be made clearer. Only after reading the manuscript a couple times did I realize that the HumanCyc pathways aren’t used until page 9, and then only for the selective-sweep data. Some more signposts in the text relating the current work to the previous one would be helpful. I would also suggest moving the introduction to the HumanCyc pathways to the point where they are used. How much data was shared between the two papers? I’m not sure from the methods exactly which new dN/dS values were (re) calculated and how many were taken from the 2018 paper. I am particularly confused by the last sentence of “Purifying selection during primate and rodent divergence” (top of page 15). There were evolutionary stats retrieved from the 2018 paper for 843 genes, and then the remaining ones needed for Recon3D were newly calculated? If that’s the case, the 2018 paper used Ensembl 75 (Feb 2014) but here they used Ensembl 85 (Jul 2016). The human genome assembly went from GRCh37 to GRCh38 in that time. I really think it would be stronger if the 2018 values were recalculated against the same assembly.

***Author’s response:*** *We have clarified in the methods and main text which data we use from Montanucci* et al *2018. We mistakenly wrote that we retrieved dN/dS values for HumanCyc genes. However, we only used the list of genes and reactions belonging to base pathways and their connectivity values (degree, in-degree and out-degree). While there are 768 overlapping genes between this dataset and the genes present in Recon 3D, we do not use the original dN/dS values calculated by Montanucci* et al. *2018. We calculated dN/dS for all genes in Recon 3D, whether they are present or not in the HumanCyc dataset.*

*As a test we compared the dN/dS values for the genes present in both datasets (n = 768). Although some values differ, the correlation between the values obtained in both studies is very high (shown below). We do not think necessary to repeat the analyses in Montanucci* et al*. 2018 and reanalyze HumanCyc dataset with a new Ensembl release.*

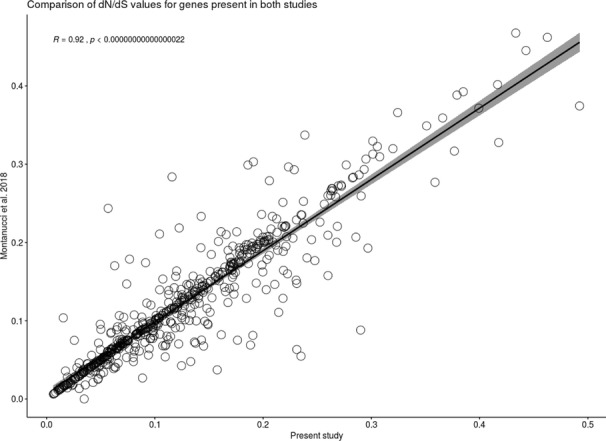



### Reviewer comment

Most of the paper is couched firmly in technical network terms with very little discussion of the underlying biology. The Discussion section, in particular, mostly reiterates the Results when it could be used to tie them together in the context of the biology. What are the causes and implications of the observed patterns of selection? I was surprised that there was no mention of metabolic flux, especially given the authors’ previous publications (Colombo et al. 2014. Evolution). Similarly, no attempt is made to connect the disparate patterns between the different evolutionary scales (divergence, complete sweep, incomplete sweep).

***Author’s response:*** *In this study we have focused on the topology of the network and not on its dynamics. Although it is possible to estimate the metabolic flux distribution for the whole human metabolic network, we argue that this approach is better suited for smaller systems, where compartmentalization or tissue-specific expression is considered. In Colombo* et al *2014, the authors selected a small and tissue-specific network (the core metabolic network of the human erythrocyte), which is very well studied and endowed with a high detail of experimental data on the kinetics of the reactions. This system had been studied with kinetic, stochastic and constraint-based models and a robust set of flux values was derived. However, there is a lack of experimentally determined values for most of enzymes and finding biologically sounded objective functions for eukaryotic cells to apply flux balance analysis (FBA) is not a trivial matter. Even if it would be extremely interesting, it is outside the scope of this paper to analyze the effect of kinetics on the evolutionary patterns of metabolic genes. However, we have commented in the main text the relationship between metabolic flux and enzyme evolution to highlight that we do not consider that effect there.*

### Reviewer comment

The authors barely touch on the fact that there is a many-to-many mapping of genes to nodes. That is, one gene can be present in multiple nodes, and one node can have multiple genes. This means that the datapoints in their statistical tests are not independent. Some genes’ selection metrics appear multiple times, and some nodes’ network metrics appear multiple times. For example, node 3.1.4.11-RXN is listed 117 times in Additional file 2: Table S6. The authors don’t state how they handle that, so I have to assume that the node appears 117 times in the pathway analysis, which over-inflates its metrics in the statistical tests. At the very least, mean selection metrics need to be computed for each node and these should be used in the various statistical analyses. However, they still won’t be independent, so first some genes and nodes might need to be removed due to redundancy. Do genes that share the same nodes tend to have similar selection metrics? Do nodes with significant gene-overlap have similar network metrics?

***Author’s response:*** *We have clarified how we decided to address the lack of one-to-one correspondence between genes and enzymatic reactions in the Methods. An enzymatic reaction can be coded by one or more genes, either as an enzymatic complex or by isozymes. Thus, if an enzymatic reaction (node) is encoded by more than one gene, that node will be associated with as many values of the selection metrics as genes is encoded by. Similarly, if the protein encoded by a gene participates in more than one enzymatic reaction, that gene will be associated with as many connectivity measures as reactions it participates in.*

*We do not calculate an average selection metric value per node or an average connectivity measure per gene due to the following reasons:*

*1) Such calculation will decrease the experimental error (within or residual variation), thus increasing artificially our statistical power. Obviously not recommended.*

*2) It is not necessarily expected that subunits encoded by different genes will have the same signature of positive selection, as they can have different functionalities. Unless there is a compensatory mechanism in the other subunit(s), only the one(s) with the function related to the phenotype under selection are expected to have the signal. Thus, averaging will remove that signature.*

*3) In the case of isozymes, their expression can be location, tissue or development specific. Thus, again it is not expected that all will have the signature of positive selection as the context of their activity can vary.*

*4) We tested whether genes detected under positive selection by a given test participate in a different number of enzymatic reactions than the rest of metabolic genes and we did not find any differences (Permutation test, p-value > 0.05 in all comparisons).*

*As reviewer 1 pointed out, the metabolic model used is simplistic and does not incorporate tissue or developmental-specific expression. Thus, we have not tried to differentiate all these possible scenarios and decided to keep all the possible gene-reaction measures. This approach is again decreasing our statistical power as a gene under positive selection can be associated with several values of degree, increasing the noise in the analyses. But it increases our confidence that the signals found are robust to overcome the noise present in the data.*

*To answer the reviewer question on whether genes that share the same nodes tend to have similar selection metrics, we performed the following comparison: for each set of reactions encoded by a given number of genes, we calculated the standard deviation (sd) of the selection metric. Then we generated a distribution by randomly sampling the same number of gene selection metric values for each set of reactions encoded by a given number of genes (number of permutations = 100) and calculating the sd. If genes that share the same nodes have similar selection metrics our expectation was to find a smaller standard deviation for the genes associated to the same nodes in comparison to randomly sampled genes. We compared the sampling interquartile range (percentile 25th–75th range) between both distributions for each selection metric (see figure below). The range of the sd distribution overlaps in all cases, but genes of the same node have more similar values than those sampled randomly as indicated by the distribution of real sd values reaching smaller values than the permutations. While this result points out that the expectations of the referee are correct, and that genes associated with the same node are more likely to have similar selection metrics, we still think that the original values and not an average should be used to avoid an artificial statistical power inflation.*

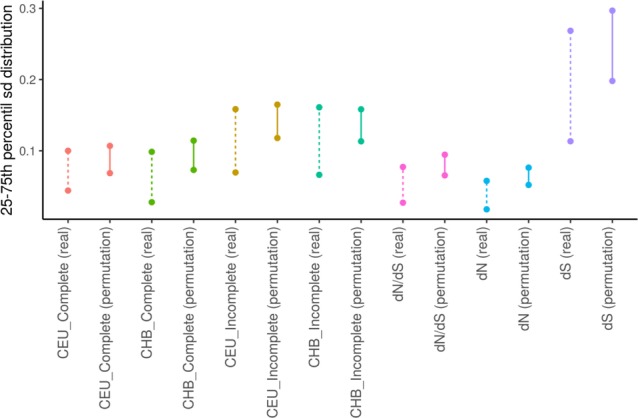



### Reviewer comment

Since the authors rely on residuals from an ordinary least-squares regression, they should verify that the residuals meet the assumptions of OLS, namely that they be homoscedastic. Otherwise, a different method like generalized linear models should be used. Regardless of the model used, the residuals need to be standardized to be comparable to each other. Otherwise, the variance of the residuals of peripheral data points with higher leverage will be smaller than the points at the center, which will affect the analyses.

***Author’s response:*** *In this study we rely mostly on nonparametric methods and calculation of p-values by permutations to minimize the effect of the deviation of parametric test assumptions. However, to satisfy the assumption of heteroscedasticity of the residuals we transformed the variables prior to applying the linear regression to control for the confounding factors mentioned. We have added the explanation in the Methods section and changed the Results and Discussion accordingly. Even though some specific results have changed, the main conclusions remain the same.*

### Reviewer comment

On page 9, the authors state that genes catalyzing the last steps in the metabolic network have higher HB values both for complete and incomplete sweeps but that the trend is weaker in CHB. However, Additional file 1: Figure S5b shows that the trend does not exist in CHB for incomplete sweeps. For complete sweeps, the trend is different, not weaker: the “top” and “intermediate” residual values look approximately the same between the two populations. Only “bottom” differs.

***Author’s response:*** *Thank you for pointing out this mistake. We have changed the text accordingly.*

### Reviewer comment

I think “omega” is a CODEML-specific thing. dN/dS is probably clearer.

***Author’s response:*** *That is correct. We have changed omega (w) for dN/dS to not confuse readers unfamiliar with CODEML-PAML terminology.*

### Reviewer comment

On page 5, the Recon3D interactions are said to be less reliable. Why?

***Author’s response*** *: Recon3D is the result of a general model of the metabolism, where specific reactions may be theoretically feasible but in practice be cell specific. In addition, it is a metabolic reconstruction and must fulfill the requirements for metabolic modelling. Therefore, there are reactions that have been included to meet that requirement that have lower confidence than reactions with a direct experimental evidence of the gene product and the biochemical reaction. We have clarified this in the Background section.*

### Reviewer comment

On page 6: Purifying selection is stronger in nodes with more connections (Fig. 1) A bit pedantic, but because they are using the residuals, I don’t think that’s the correct interpretation. For example, the “25–50%” connectivity class all average around 0, meaning that their dN/dS values tend to be as expected given their sequence characteristics. This kind of language should be checked throughout the manuscript.

***Author’s response:*** *We agree that using the residuals instead of the original dN/dS values makes the interpretation confusing, but our interpretation of the results is correct, once we exclude the effect of the confounding factors, purifying selection (as estimated by dN/dS) is stronger in nodes with more connections. We have rephrased that sentence to clarify it and we have checked the language used in the rest of the manuscript. We have added the* Figure 1 *with the original values in the* Additional file 1 *to help with the visualization of the results (see* Additional file 1*:* Figure S4).

**Reviewer comments to Authors:** The authors have satisfactorily addressed my concerns. I would just like to clarify that I did not suggest that a flux-based analysis be performed. I agree that it would be out of scope and potentially infeasible at this scale. I suggested it as an important topic that should be discussed somewhere in the manuscript to help explain some of the observed patterns of selection (that is, the “network” is a mathematical convenience, but the flux or information flow that embodies that network is of immediate biological, and therefore evolutionary, relevance). In any case, this has now been resolved by the authors.

## Additional files

Additional file 1: **Figure S1.** Reaction graph generated from the human metabolic network reconstruction Recon3D. **Figure S2.** Distribution of the selection estimates calculated for genes with 1:1 orthologs in the 6 species (Human, Chimpanzee, Gorilla, Orangutan, Mouse, and Rat) in the global metabolic network. **Figure S3.** Correlation matrices between variables. **Figure S4.** Relationship between selection estimates and connectivity (degree, in-degree and out-degree) in the global metabolic network. **Figure S5.** Relationship between selection estimates and position. **Figure S6.** Number of genes under positive selection in each functional pathway of the global metabolic network. **Figure S7.** Distribution of the number of enzymatic reactions carried by a given gene. (DOCX 1804 kb)

Additional file 2: **Table S1.** Reaction graph. List of edges of the directed reaction graph generated formed by the giant connected component of Recon3D. **Table S2.** Genes under positive selection in the global metabolic network. **Table S3.** Genes under recent positive selection in individual metabolic pathways. **Table S4.** Connectivity of metabolic genes under positive selection compared to the rest of metabolic genes in individual metabolic pathways. **Table S5.** Global metabolic network giant connected component gene/reaction information. **Table S6.** Individual metabolic pathways gene/reaction information. (XLSX 4140 kb)

## Data Availability

All data generated and analyzed during this study are included in this published article and its additional files.

## Abbreviations

CDS: Protein-coding sequence
CEU: Utah residents with Northern and Western European Ancestry
CHB: Han Chinese from Beijing, China
FDR: False Discovery Rate
HB: Hierarchical Boosting
Kya: Thousand years ago
LRT: Likelihood ratio test
PIN: Protein-protein interaction network
sd: standard deviation
YRI: Yoruba from Ibadan, Nigeria
