# Supplementary material for: Gene connectivity and enzyme evolution in the human metabolic network
Source: Biol Direct. 2019 Sep 3;14:17. doi: 10.1186/s13062-019-0248-7 (PMC6724310; doi:10.1186/s13062-019-0248-7)
Supplement: Supplementary file 1 — Figure S1. Reaction graph generated from the human metabolic network reconstruction Recon3D. Figure S2. Distribution of the selection estimates calculated for genes with 1:1 orthologs in the 6 species (Human, Chimpanzee, Gorilla, Orangutan, Mouse, and Rat) in the global metabolic network. Figure S3. Correlation matrices between variables. Figure S4. Relationship between selection estimates and connectivity (degree, in-degree and out-degree) in the global metabolic network. Figure S5. Relationship between selection estimates and position. Figure S6. Number of genes under positive selection in each functional pathway of the global metabolic network. Figure S7. Distribution of the number of enzymatic reactions carried by a given gene. (DOCX 1804 kb) [file 13062_2019_248_MOESM1_ESM.docx]

Supplementary Figure 1. Reaction graph generated from the human metabolic network reconstruction Recon3D. Nodes represent reactions and are connected by directed links based on shared metabolites. After removing currency metabolites, we obtain one giant connected component comprising 88.72% of the reactions, 145 small connected components (1.37% of the reactions), and 821 isolated reactions (7.75%). Only the giant connected component (global metabolic network) was considered for the analyses. Size and color of nodes correlate with node out-degree. Plotted with graph-tool [1].


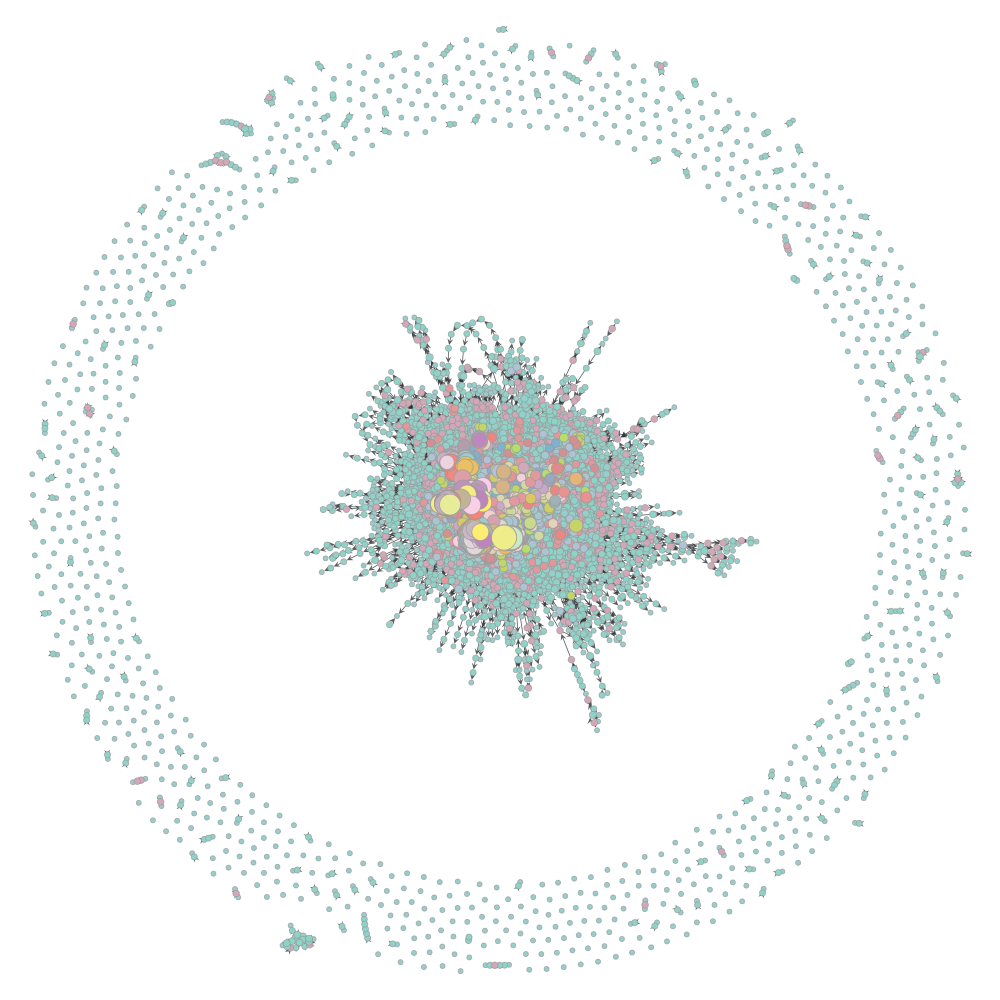


Supplementary Figure 2. Distribution of the selection estimates calculated for genes with 1:1 orthologs in the 6 species (Human, Chimpanzee, Gorilla, Orangutan, Mouse, and Rat) in the global metabolic network. a) dN/dS; b) dN = number of nonsynonymous substitutions per nonsynonymous site; c) dS = number of synonymous substitutions per synonymous site.

a)


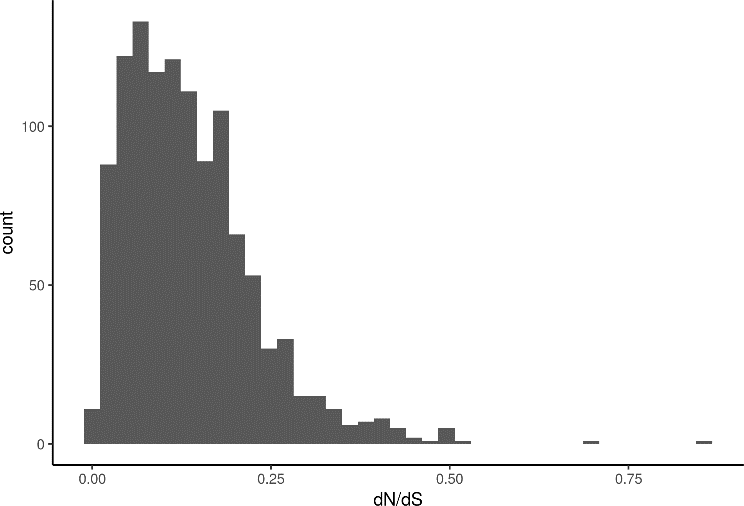


b)


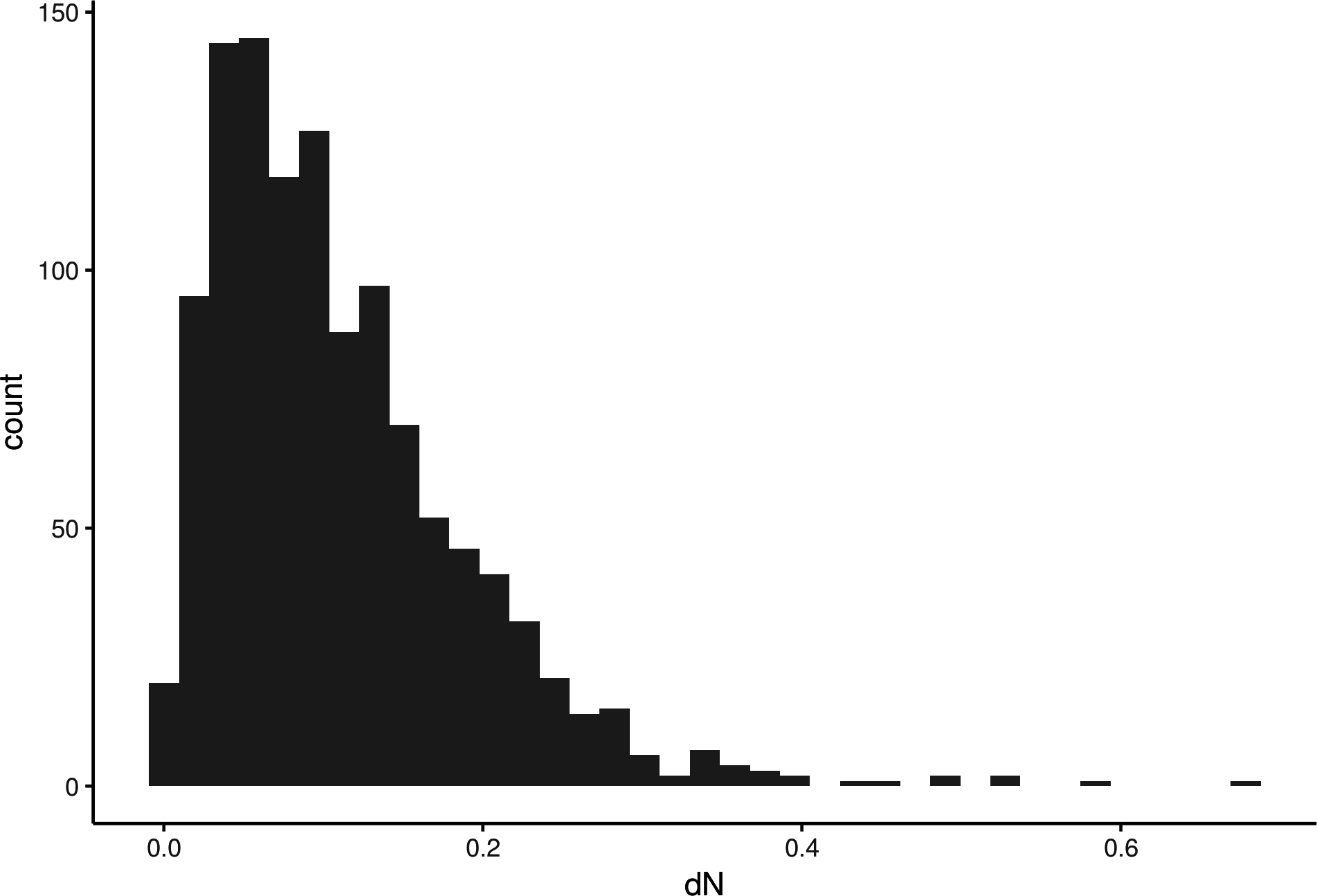


c)


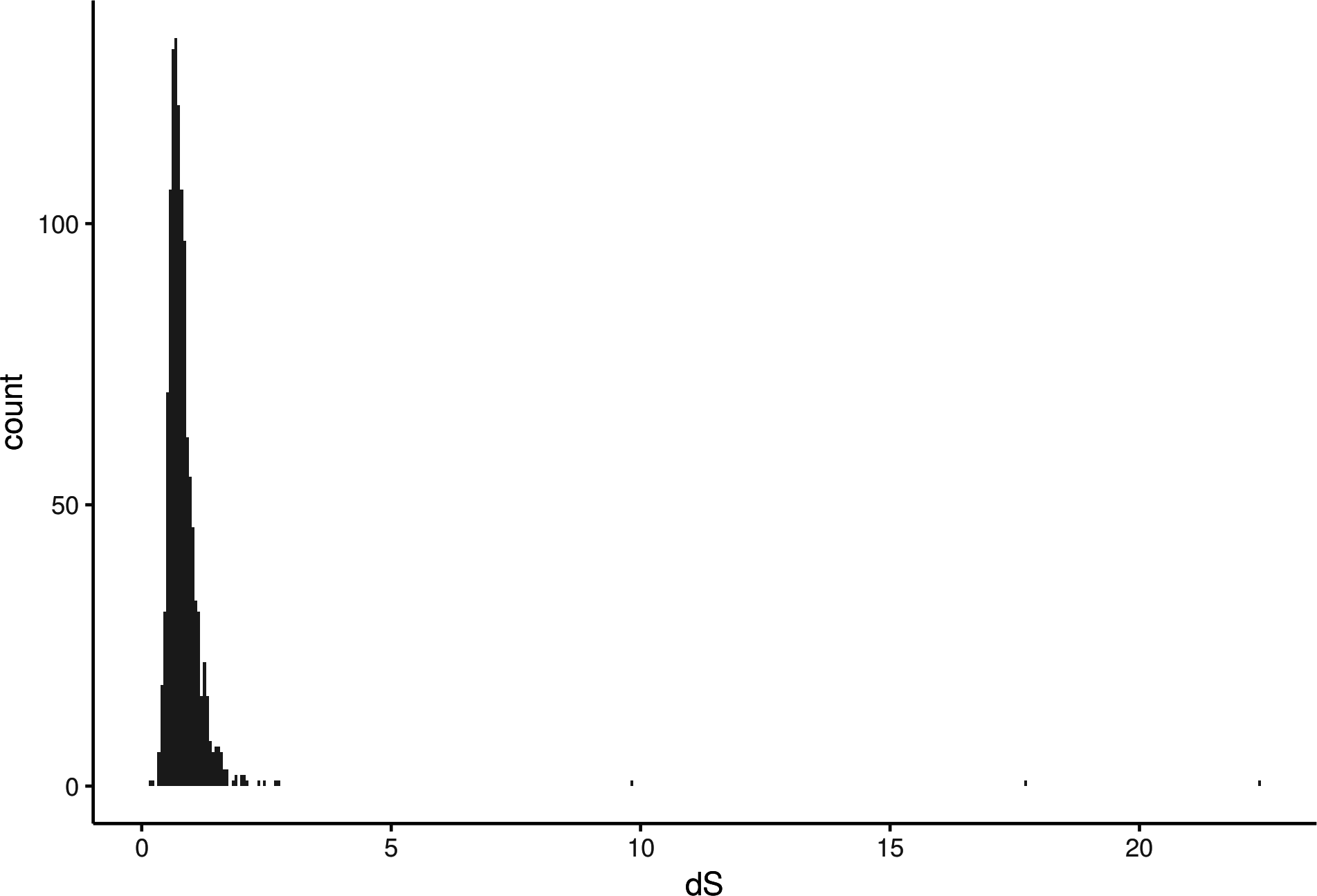


Supplementary Figure 3. Correlation matrices between variables. a) Correlation matrix with selection estimates calculated in CEU and CHB human populations (Complete and Incomplete boosting) and during mammal and rodent divergence (dN/dS, dS, and dN) in the global metabolic network; b) Correlation matrix with selection estimates calculated in CEU and CHB human populations (Complete and Incomplete boosting) in individual metabolic pathways. GENE_LEN = CDS length, GC = GC content, ENC = effective number of codons (used as a proxy for codon bias), NumReactions = number of enzymatic reactions carried by a given gene. Spearman’s correlation coefficients are shown for correlations with p-value < 0.05. Size and color of the square depends on the value of the correlation coefficient.

a)


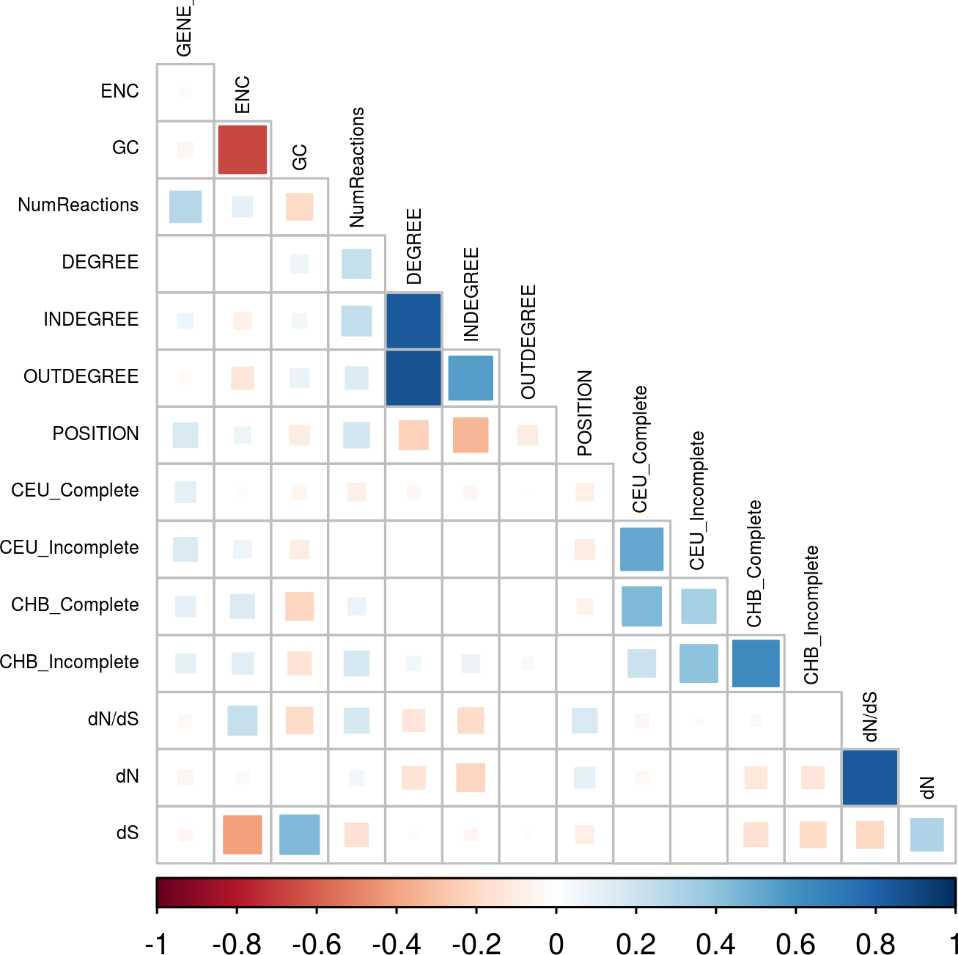


b)


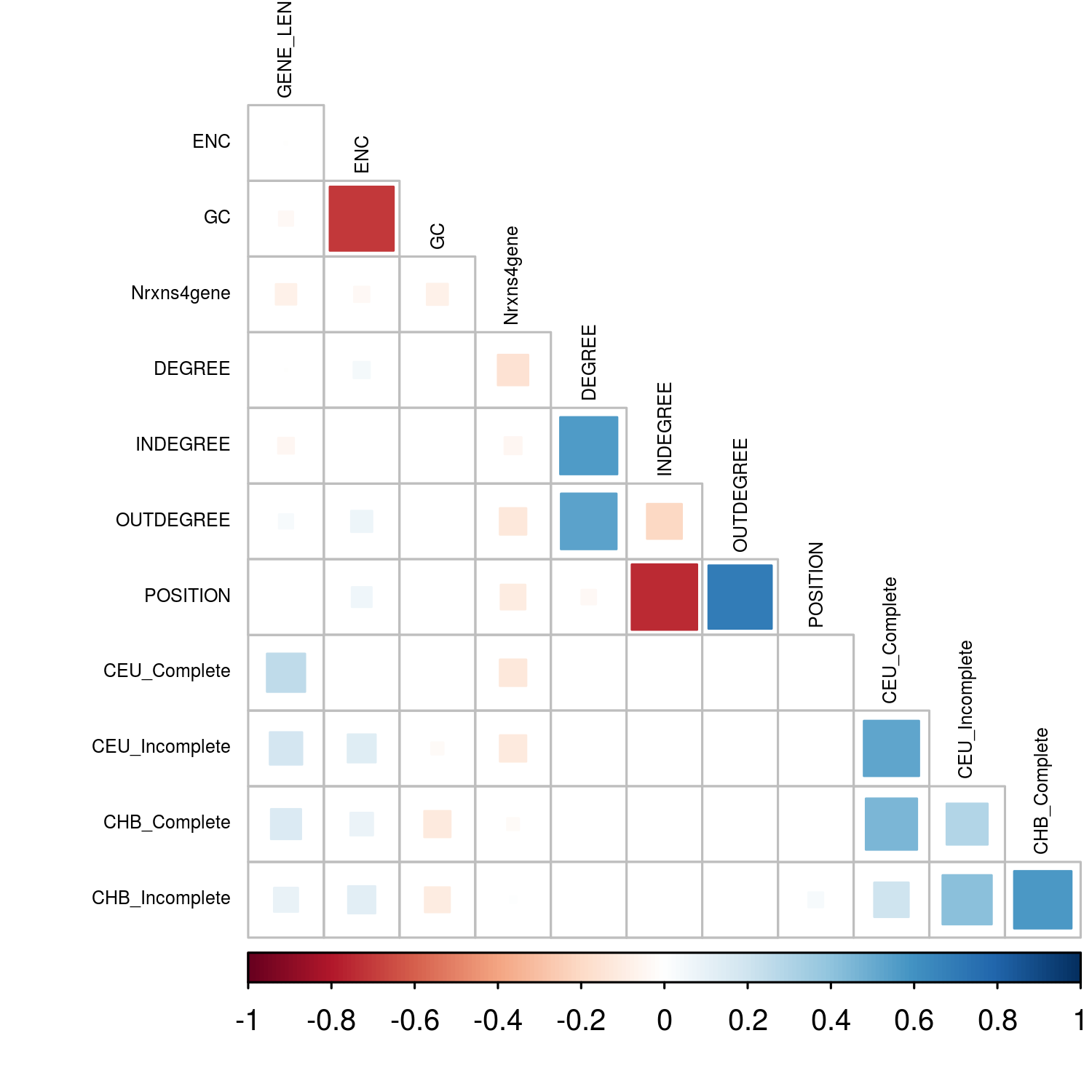


Supplementary Figure 4. Relationship between selection estimates and connectivity (degree, in-degree and out-degree) in the global network. Nodes were classified by the 25^th^, 50^th^, and 75^th^ percentile. a) mean ± standard error of dN/dS original values. For b) dN, c) dS, d) Complete CEU, e) Complete CHB, f) Incomplete CEU and g) Incomplete CHB is plotted for each group the mean ± standard error of the residuals of the linear regression controlling for genomic variables (CDS length, codon bias, and GC content) after transformation. Boosting scores are measured as the maximum score per gene. Wilcoxon test p-values for pairwise comparisons are adjusted by FDR (ns: p > 0.05; *: p <= 0.05; **: p <= 0.01; ***: p <= 0.001; ****: p <= 0.0001).

a)


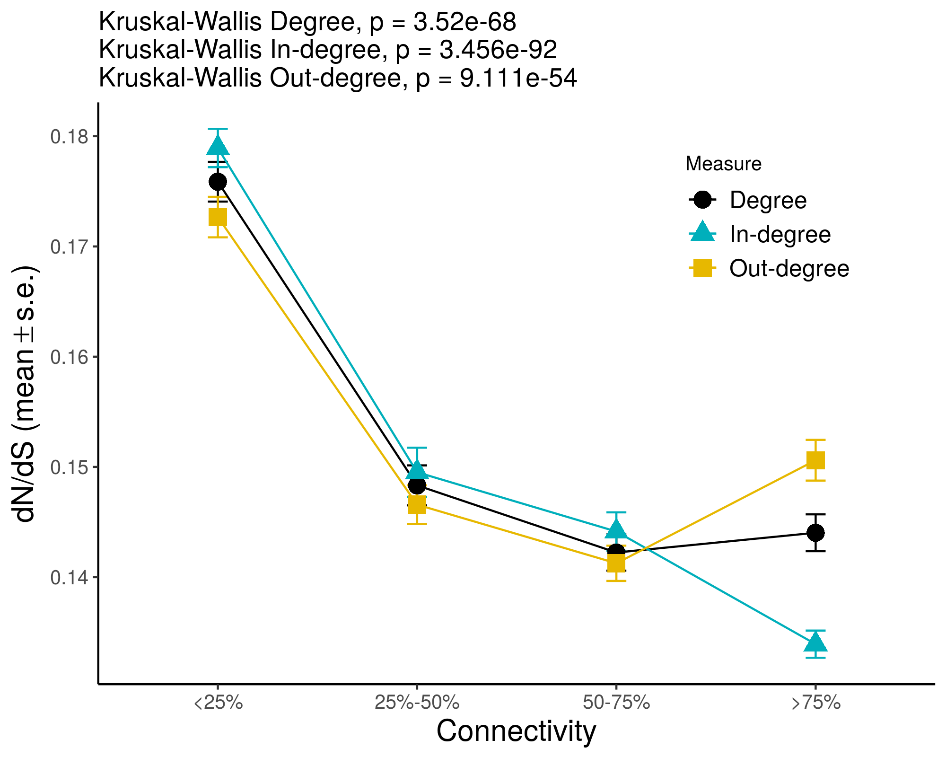


b)


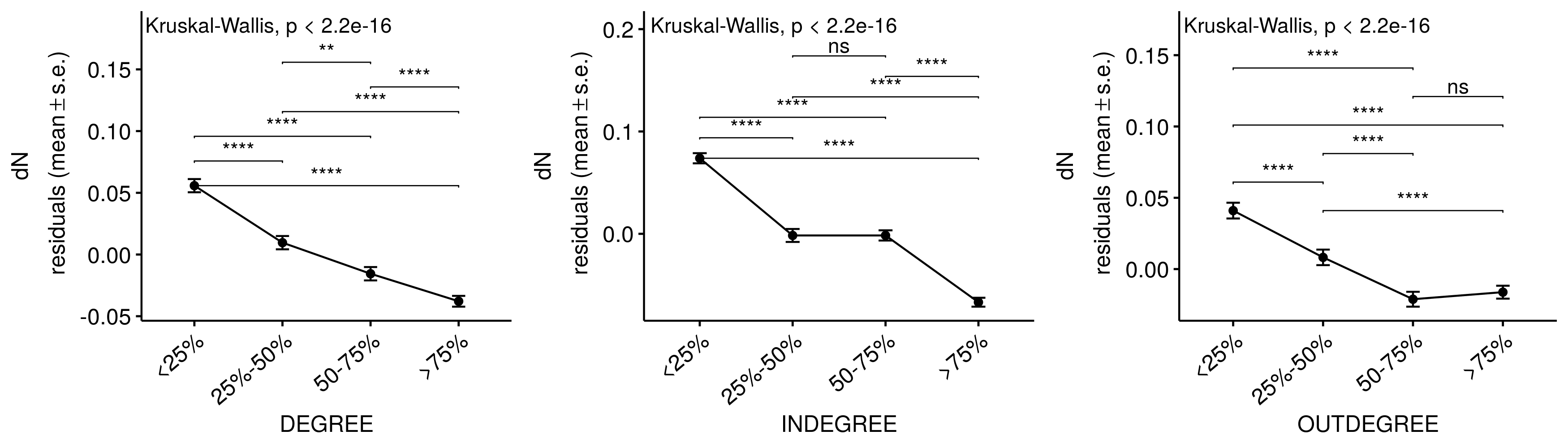


c)


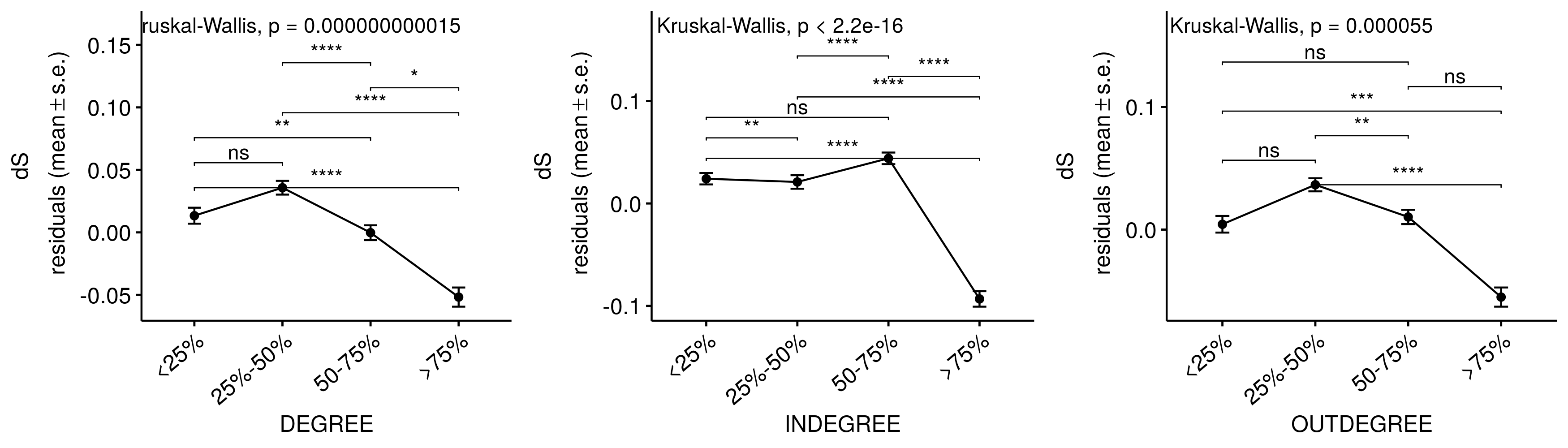


d)


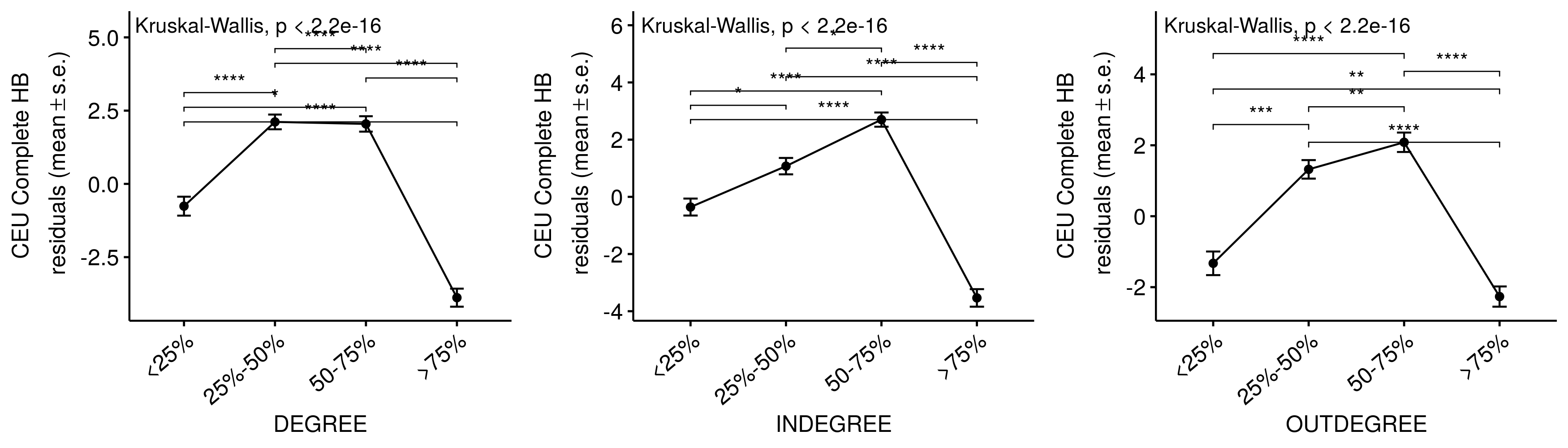


e)


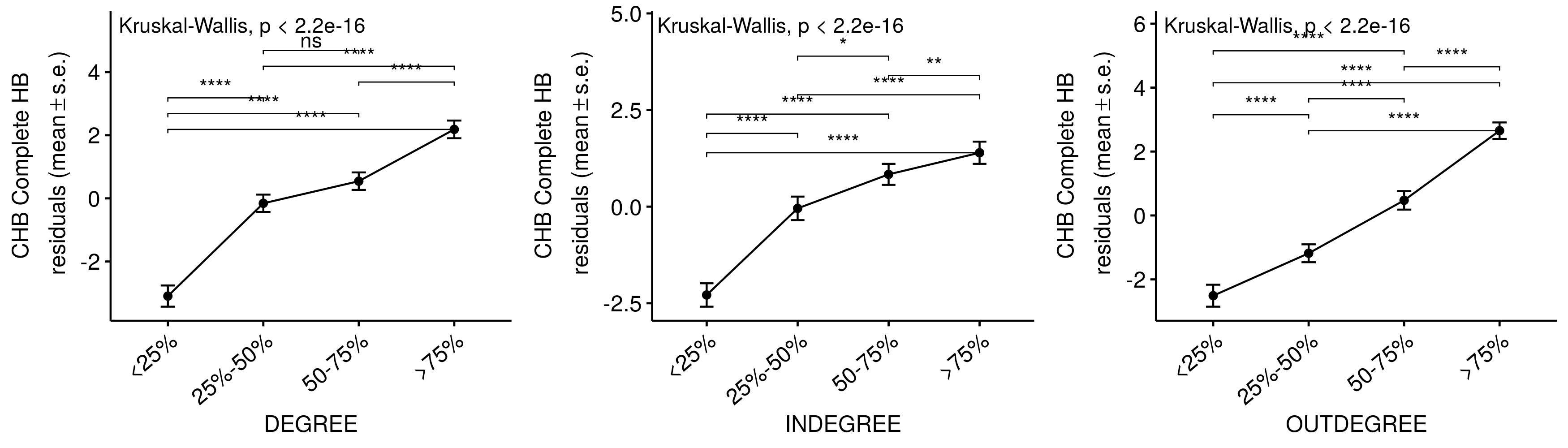


f)


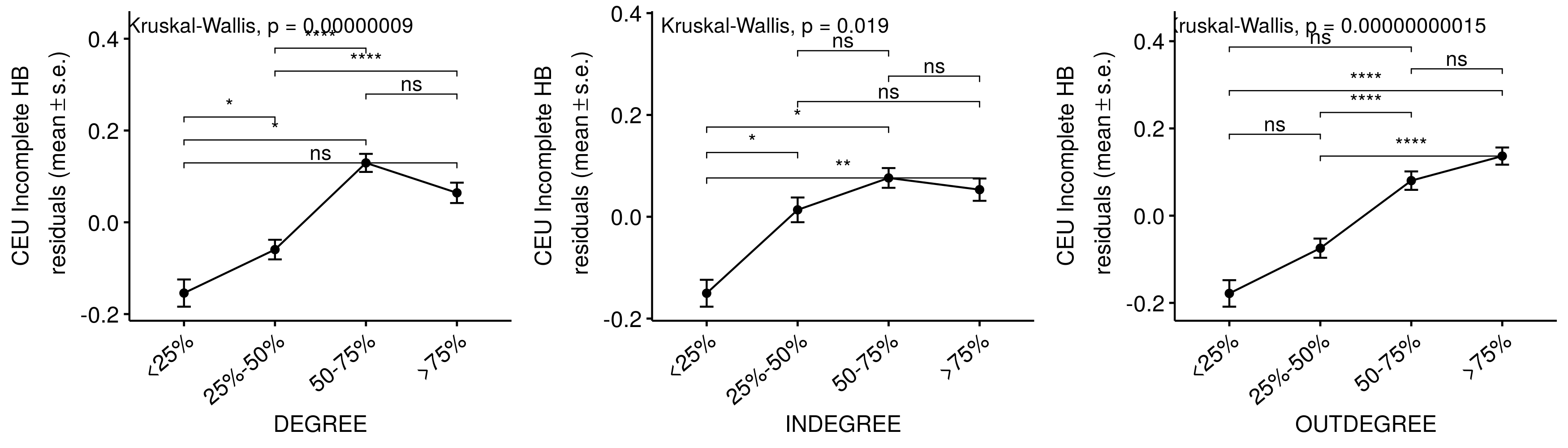


g)


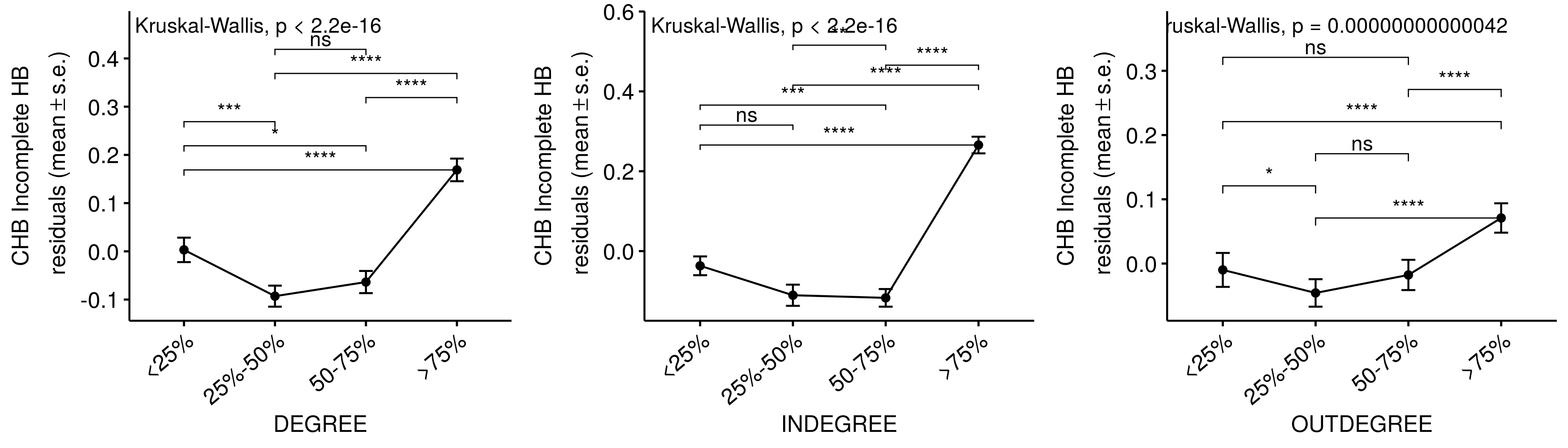


Supplementary Figure 5. Relationship between selection estimates and position. Node position within the pathway was classified as: top, intermediate or bottom. a) dN/dS, dN, and dS; b) CEU Complete, CHB Complete, CEU Incomplete, and CHB Incomplete in the global metabolic network. c) CEU incomplete in individual metabolic pathways. Mean ± standard error of the residuals of a linear regression controlling for genomic variables (CDS length, codon bias, and GC content) after transformation is plotted for each category. Wilcoxon test p-values for pairwise comparisons are adjusted by FDR (ns: p > 0.05; *: p <= 0.05; **: p <= 0.01; ***: p <= 0.001; ****: p <= 0.0001). d) Number of genes under positive selection in a given test in each position compared to the rest of metabolic genes. Pearson's Chi-squared test M7/M8, Χ² = 1200, p-value = 0.0005; Pearson's Chi-squared test CEU Complete, Χ² = 16, p-value = 0.01; Pearson's Chi-squared test CEU Incomplete, Χ² = 14, p-value = 0.002. Square size is proportional to the contribution of each category to the total Χ² score indicated by the Pearson residuals. Positive values (in blue) indicate that the number of selected genes in category is higher than expected whereas negative values (in red) indicate that the number of selected genes is lower than expected when compared to the total number of metabolic genes. Only comparisons with p-value < 0.05 are shown (simulated p-value based on 2000 replicates).

a)


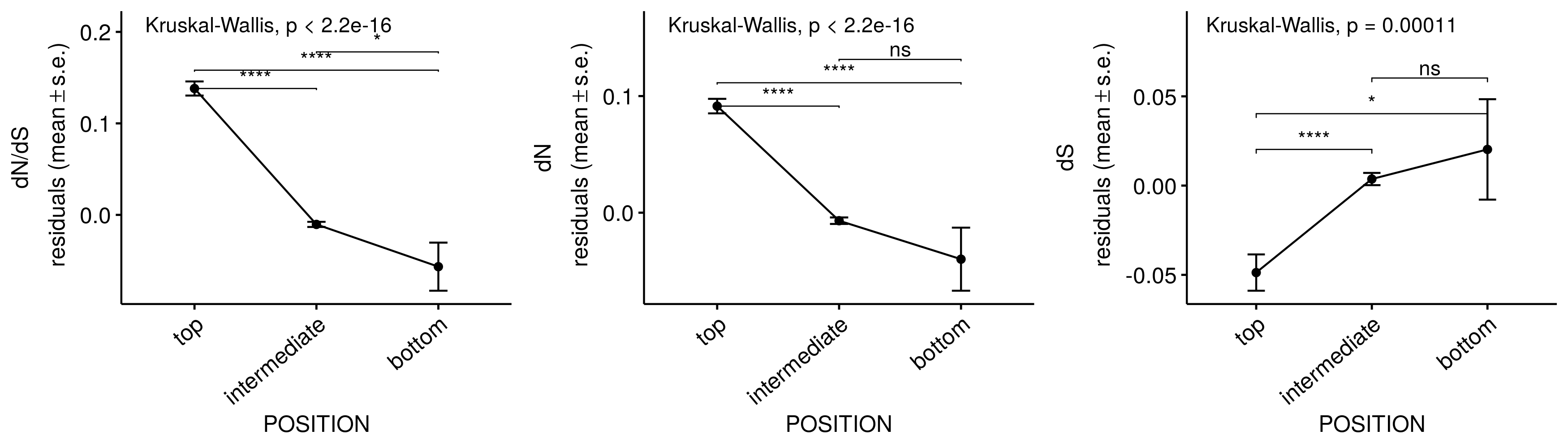


b)


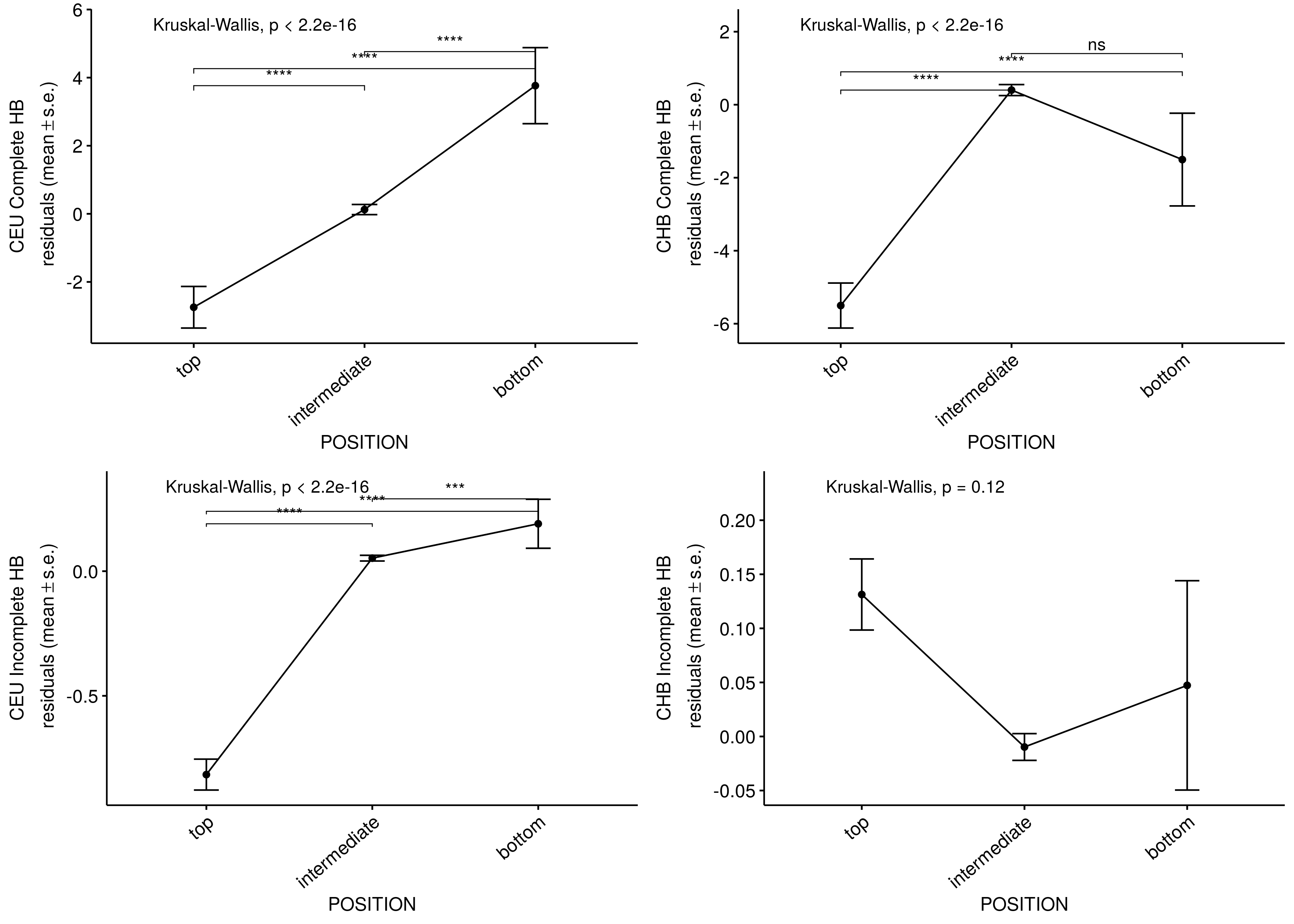


c)


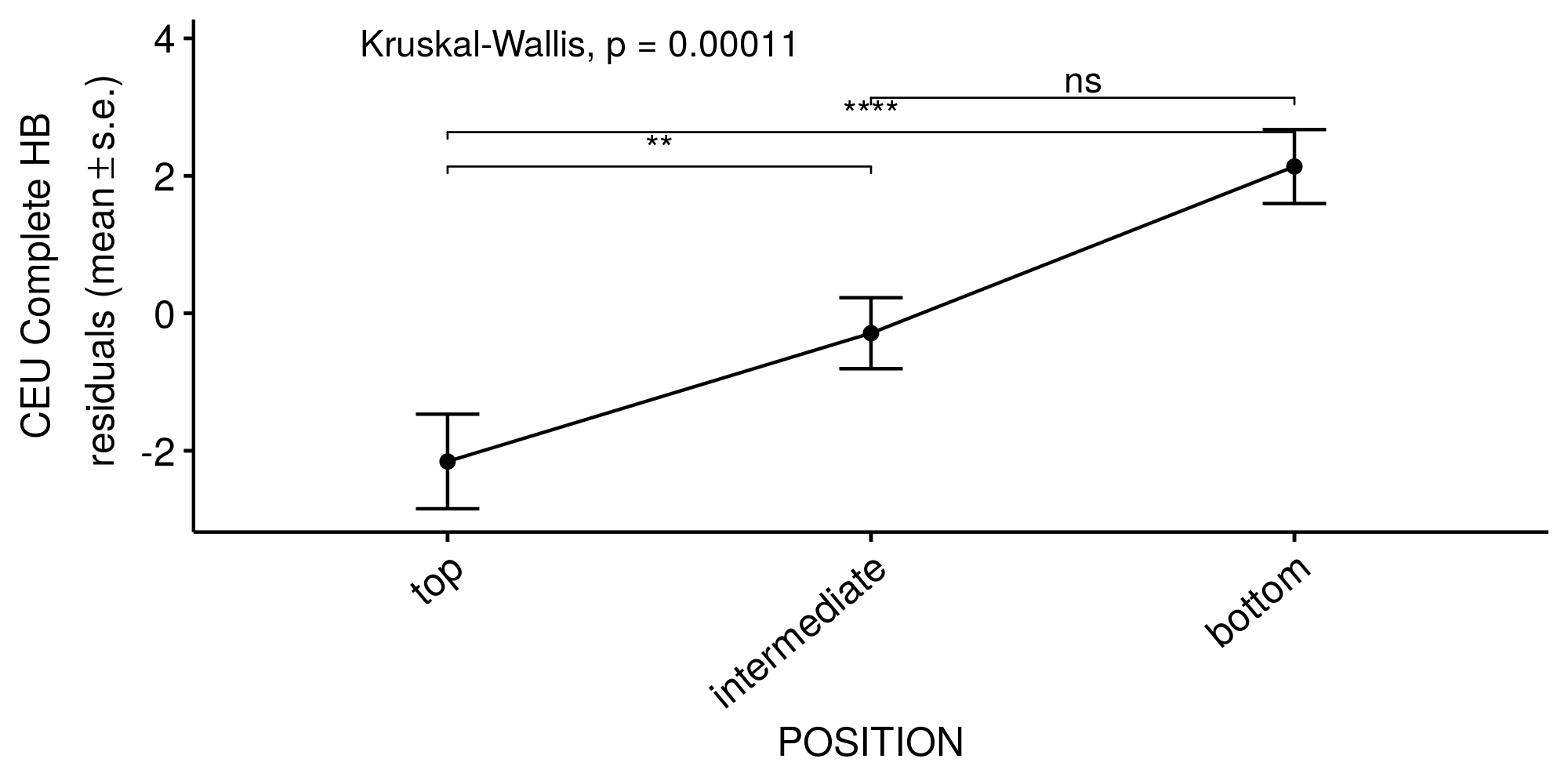


d)


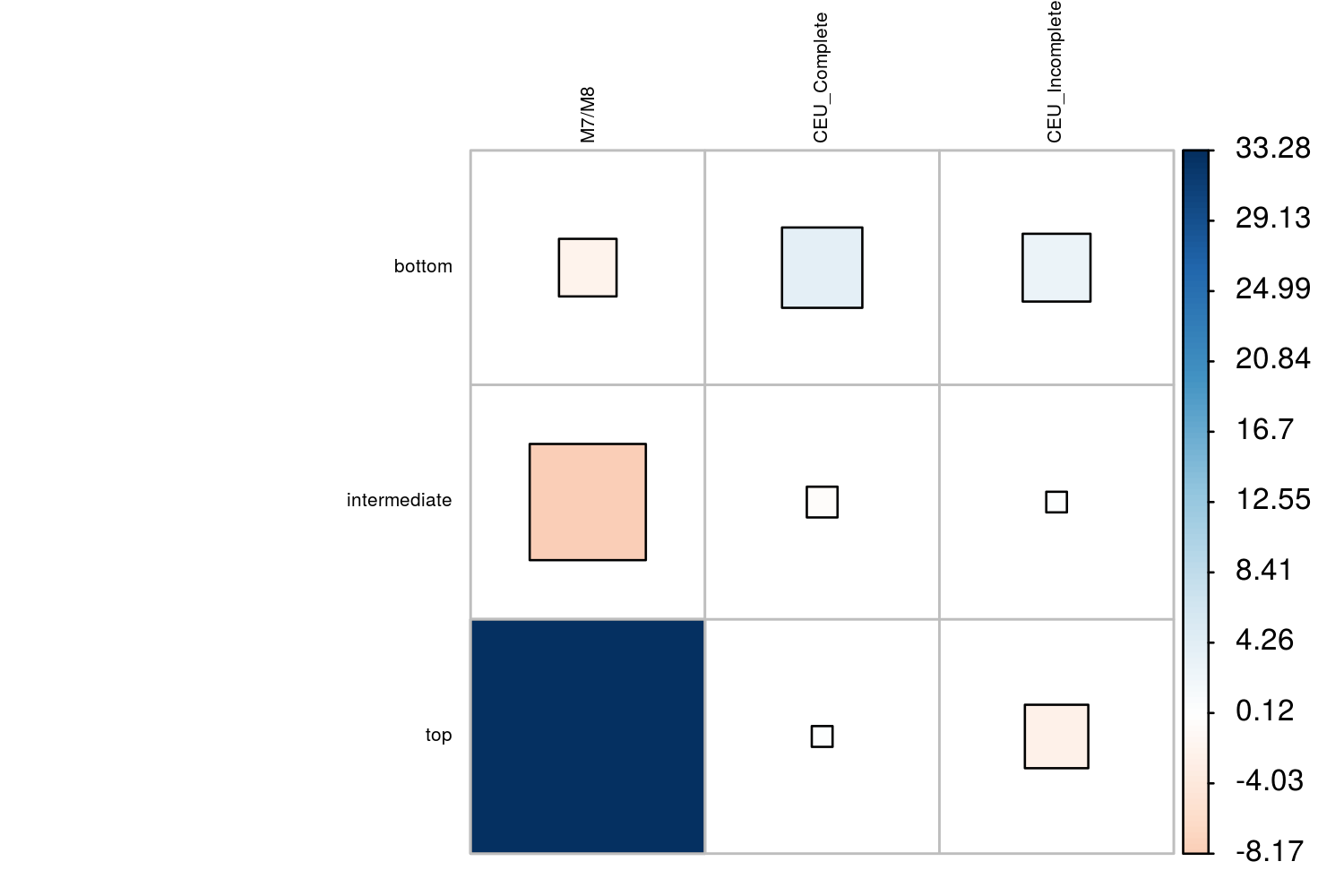


Supplementary Figure 6. Number of genes under positive selection in each functional pathway of the global metabolic network. Pearson's Chi-squared test M7/M8, Χ² = 700, p-value = 0.0005; Pearson's Chi-squared test Test 2, Χ² = 260, p-value = 0.003; Pearson's Chi-squared test CEU Complete, Χ² = 250, p-value = 0.03; Pearson's Chi-squared test CHB Complete, Χ² = 620, p-value = 0.0005; Pearson's Chi-squared test CEU Incomplete, Χ² = 330, p-value = 0.0005; Pearson's Chi-squared test CHB Incomplete, Χ² = 360, p-value = 0.0005. Square size is proportional to the contribution of each category to the total Χ² score indicated by the Pearson residuals. Positive values (in blue) indicate that the number of selected genes in category is higher than expected whereas negative values (in red) indicate that the number of selected genes is lower than expected when compared to the total number of metabolic genes. Simulated p-value based on 2000 replicates.


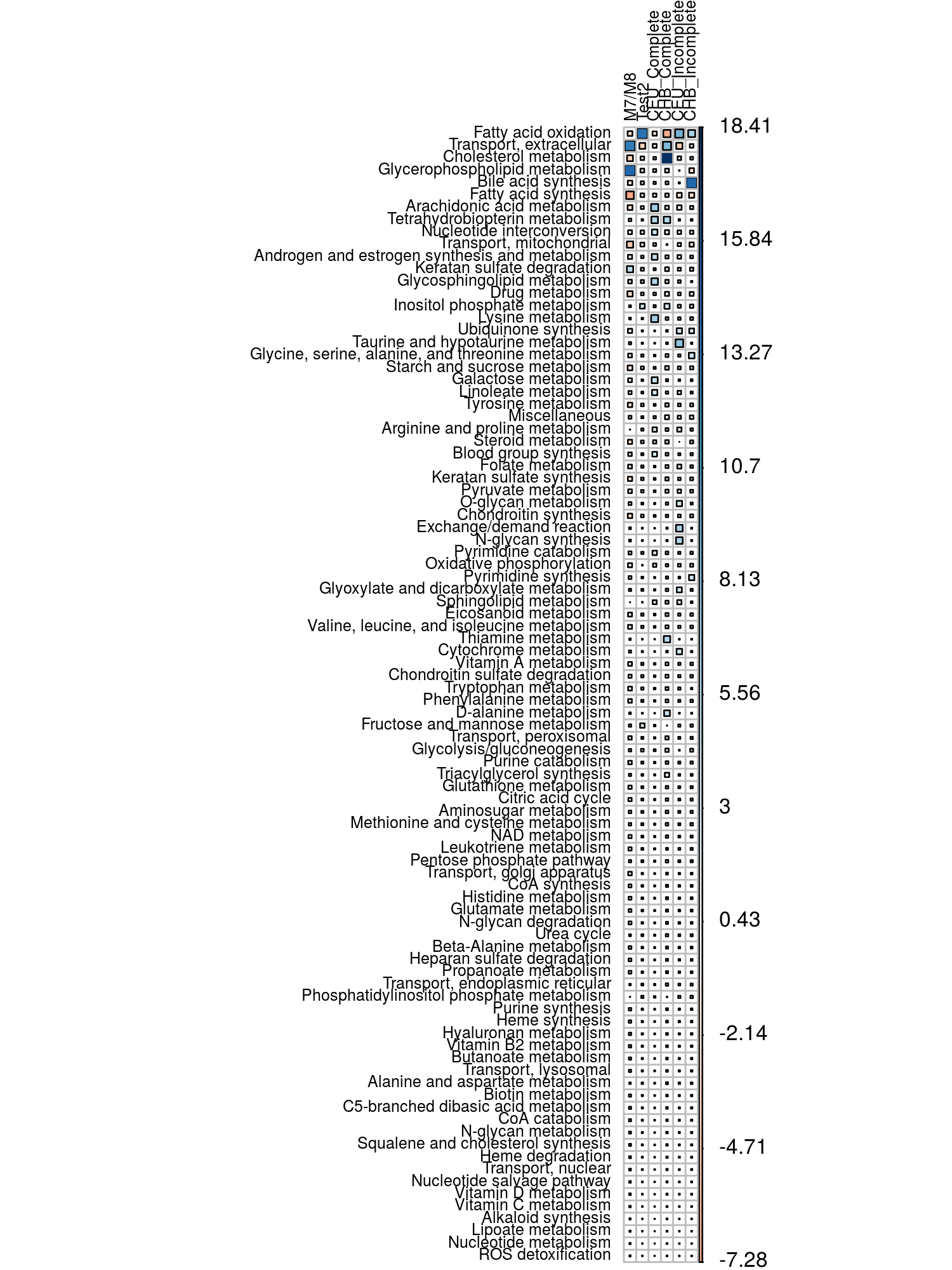


Supplementary Figure 7. Distribution of the number of enzymatic reactions carried by a given gene. On average, a gene participates in 7.44 reactions (dashed line).


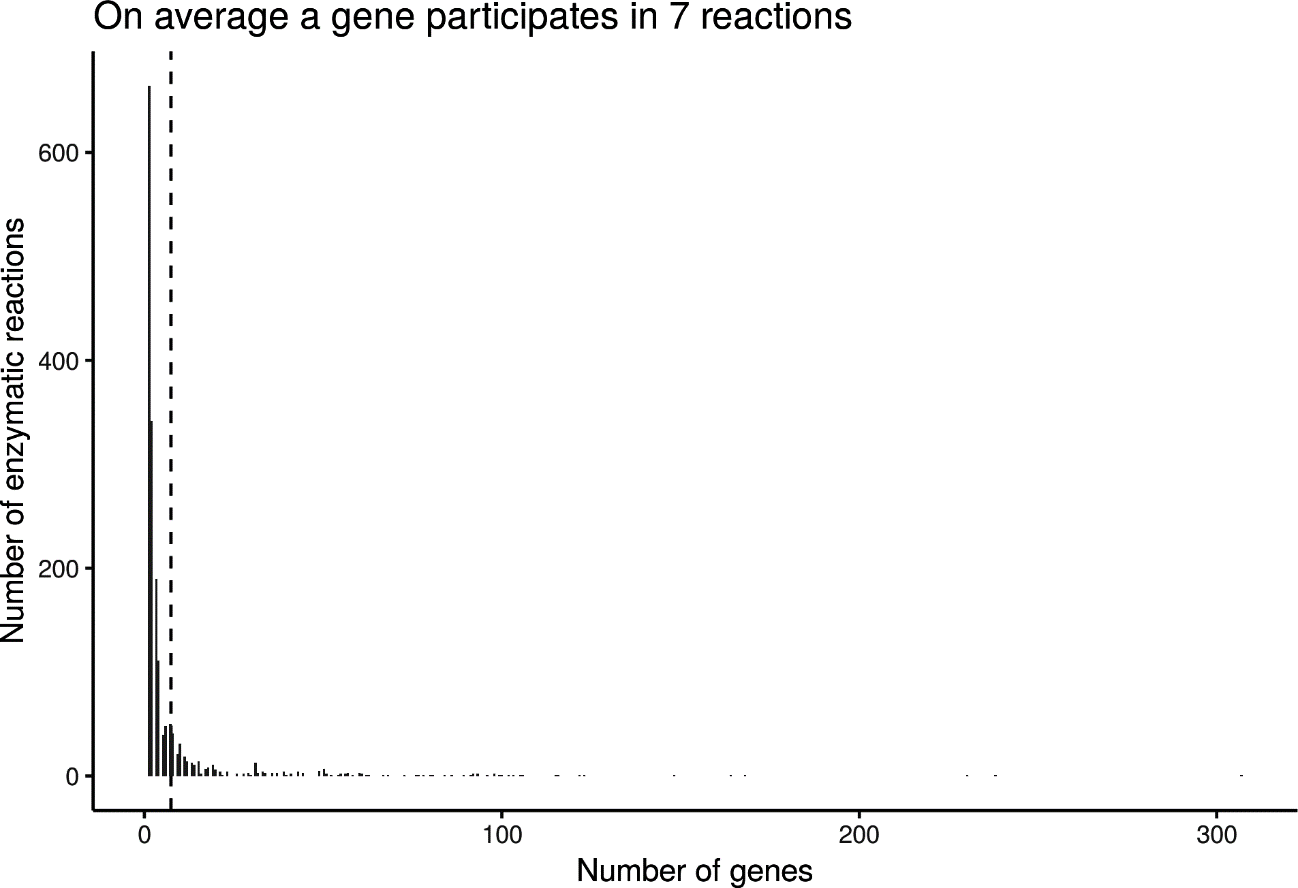


**References**

1. Tiago PP. The Graph-tool Python Library. figshare; 2017.
